# Cough hypersensitivity risk increases during heatwaves and can be reduced by inhalation of alkaline aerosols of endogenous ions

**DOI:** 10.1038/s41598-026-68025-4

**Published:** 2026-09-15

**Authors:** B. Button, A. Edwards, K. F. Chung, H. Dang, M. Gutay, N. Griffin, J. Gerenza, Linying Wang, H. Abubakar-Waziri, D. B. Bhatta, D. A. Ausiello, D. Li, B. Alotaibi, K. K. Altassan, D. A. Edwards

**Affiliations:** 1 Department of Biochemistry and Biophysics, University North Carolina, Chapel Hill, USA; 2 Department of Biomedical Engineering, Boston University, Boston, USA; 3 National Heart and Lung Institute, Imperial College London, London, UK; 4 National Key Laboratory of Earth System Numerical Modeling and Application, Institute of Atmospheric Physics, Chinese Academy of Sciences, Beijing, China; 5 SC Therapeutics, Boston, USA; 6 Massachusetts General Hospital, Boston, USA; 7 Harvard Medical School, Boston, USA; 8 Department of Earth and Environment, Boston University, Boston, USA; 9 College of Medicine, King Saud University, Riyadh, Saudi Arabia; 10 Department of Family and Community Medicine, College of Medicine, King Saud University, Riyadh, Saudi Arabia; 11 Center for Nanomedicine, Johns Hopkins University Medical School, Baltimore, MD USA

**Keywords:** Diseases, Environmental sciences, Health care, Medical research

## Abstract

Heat wave burden is rapidly rising and exacerbating human respiratory illnesses. Yet, how to protect human airways, short of deep and sustained reductions in greenhouse gas emissions, remains unclear. Here, we find, by theoretical, in vitro experimental, and human clinical studies, that heatwave exposure elevates risks of cough hypersensitivity, which can be reversed by the inhalation of alkaline hypertonic divalent salts (HDS). We find that upper-airway mucosal collapse occurs on exposing human bronchial epithelial (HBE) cell cultures to an arid heatwave atmosphere (35 ºC, 30% RH), resulting in elevated (p < 0.05) expression of genes associated with cough hypersensitivity, including TRPV4, PIEZO1, P2X3R and ENaC subunit proteins α and β (SCNN1A, SCNN1B) post 8 h—while not post 3 h—relative to baseline. We find by continuum mechanic analysis, and confirm experimentally in HBE cell cultures, that airway mucosal collapse reverses for greater than 3 h post deposition of HDS (magnesium) aerosols with pH above the cysteine pKa (approximately 8.3), while not at pH 7 or 8, nor by hypertonic saline at any pH. In a randomized, double-blind, placebo controlled study (REACH) in Riyadh, Saudi Arabia, HDS pH 9 aerosols reduced daily cough bout rate relative to placebo by 59% (p = 0.03) after 2 weeks treatment (n = 6), while, in a preceding RCC study, HDS pH 8 aerosols reduced cough bout rate by only 4% (p > 0.05) (n = 7). Alkaline HDS aerosols may be protective of human respiratory health with global warming.

**Supplementary Information:**

The online version contains supplementary material available at https://doi.org/10.1038/s41598-026-68025-4.

## Introduction

Frequency of extreme weather events is rising with global warming^1^ and worsening symptoms of respiratory illness, including asthma^2^, chronic obstructive pulmonary disease (COPD)^3^, influenza^4^, and chronic cough^5^. Impacting urban more than rural settings^6^, extreme heat and cold waves^7^ are hastening the atmospheric risks of global warming for inner city children, who are among asthmatics most at risk of asthma morbidity and mortality^8^.

Hot and cold air, similar to air at altitudes too high for survival of most plant matter^9^, expose human airways to high vapor pressure deficit (VPD)^10^, a measure of atmospheric aridity that is defined by the difference between water saturation pressure and vapor pressure at a given temperature, pressure and relative humidity^11^. At VPD levels (1.5 kPa) above which most plant leaves wilt^12^, airway mucus rapidly thins and collapses onto cilia^10^. Above VPD levels (3.0 kPa) at which most heat-resistant plants die^13^, airway mucosa compress airway epithelial cells, promoting the acute secretion of inflammatory cytokines, including IL6, IL33, and TNFα^10^, and triggering an inflammatory cascade implicated in the pathogenesis of chronic respiratory disease^14^.

**Fig. 1:**
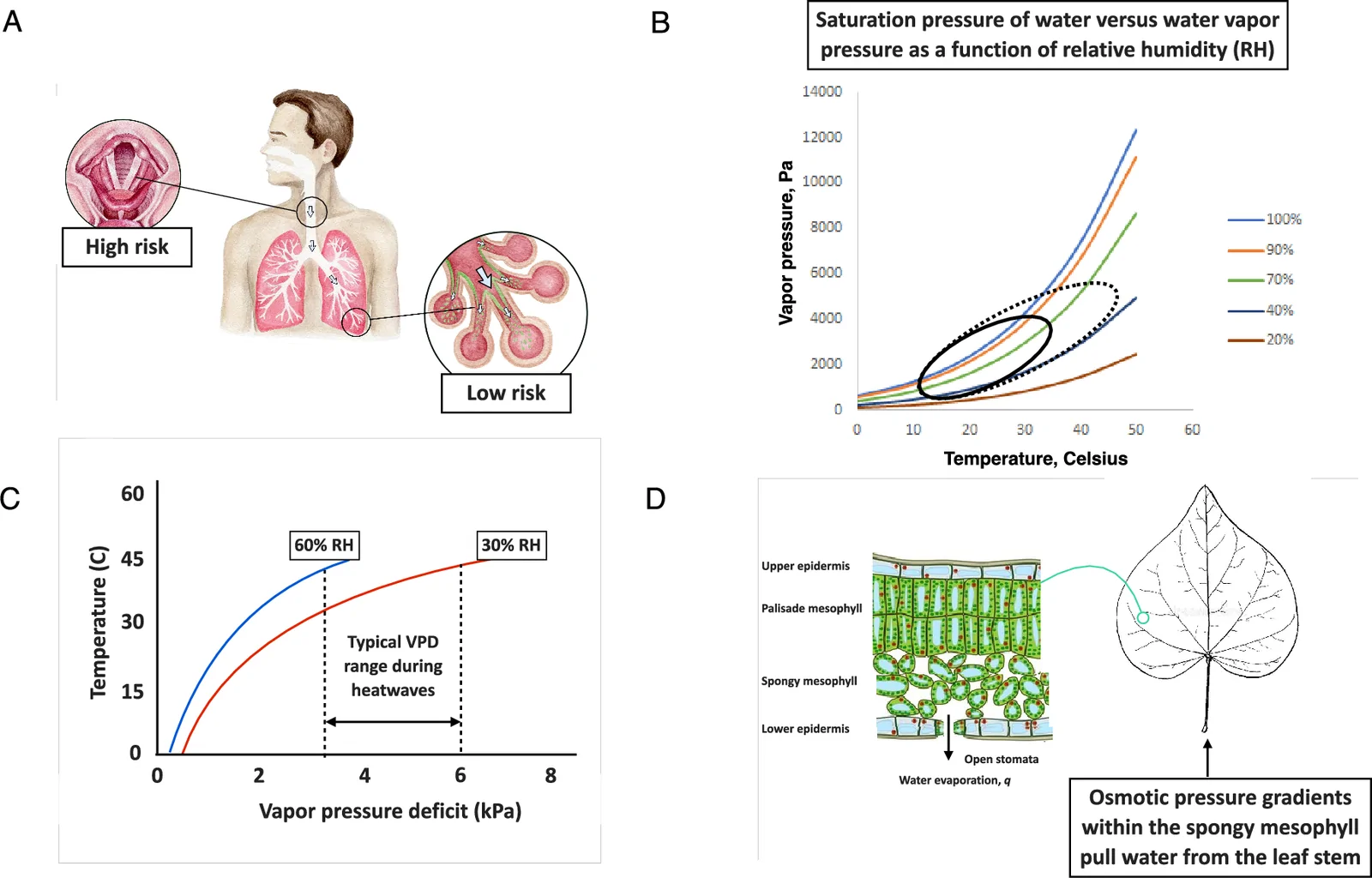
How heatwave atmospheres dehydrate human airways. (**A**) Owing to proximity to the atmosphere, mucosal collapse and dehydration risk associated with heatwave exposure is greatest in the laryngeal and tracheal regions of human airways. Original artist drawing by Heejin Gregory. (**B**) Vapor pressure deficit (VPD), the difference between water vapor pressure and saturation pressure, rises nearly exponentially with temperature at fixed RH, as depicted here on the basis of the Clausius Clapeyron Equation. The shift of temperature ranges for Continental Europe and the United States (black ellipse) over the last several decades (dotted ellipse) has therefore increased average VPD more rapidly average temperature. (**C**) Illustration of typical range of atmospheric VPD using the Clausius Clapeyron Equation, from the 3 kPa value characteristic of the June 2026 European heatwave (moderate RH range) to the extreme 6 kPa VPD characteristic of the 10 hottest cities in the United States over the last 40 years (Supplemental Information). (**D**) Illustration by the authors of the phenomenon of evapotranspiration, by which water evaporates from the spongy mesophyll of a plant leaf to promote an osmotic pressure gradient that pulls water from the leaf stem while driving inflammatory stresses at high VPD,^12,13^ governs water evaporation from human airway mucosa^10^, such that human airways face similar threats as crops with rising VPD over the course of the twenty-first century^1^.

Atmospheric aridity or VPD levels are rising nearly exponentially relative to global temperature rise^11^, with even greater elevation during heatwaves. While average global VPD rose approximately 0.05 kPa from 2000 to 2020^9^, during the recent European heatwave of June 18 to June 30, 2026, VPD rose approximately 3-fold in Paris and Madrid relative to previous 35 year averages of 0.9 ± 0.1 kPa (Paris) and 0.9 ± 0.01 kPa (Madrid) to 2.6 ± 0.9 kPa (Paris) and 3.3 ± 0.6 kPa (Madrid) (Supplemental Information). Lifetime exposure to heatwaves is predicted to increase approximately 10 to 20 fold in Brussels in 1.5 ºC to 3.5 ºC average global temperature rise scenarios^1^, devastating global crop yields, while with undetermined human airway inflammatory consequences (Fig. 1).

Mechano-sensitive ion channels (MSC) play a prominent role in the inflammatory response of human airways to the mechanical compression associated with heatwave aridity exposure^15^. MSCs such as TRPV4^16^, PIEZO1^17^, and ENaC^18^ respond to compression by elevating the secretion of ATP^19^ and IL6^20^ in the case of TRPV4, chemokine secretion such as CXCL2^21^ in the case of PIEZO1, and sodium ion permeability^22^ in the case of ENaC^23^. Up- and down-regulation of these and other MSCs, including CFTR and AQP5, is common to conditions of cough hypersensitivity^24^, and accompanies heightened tendency for cough^25^ and bronchospasm^26^.

Airway epithelial cell compression in extreme atmospheres occurs by elevation of osmotic pressure in airway surface liquid (ASL), a principal contributor to which is globular protein^27^. Over a hundred globular proteins diffuse within ASL^28^, moving from mucus into the periciliary layer (PCL) given their small size (20 to 200 kDa) relative to the mesh size between tethered mucins (MUC1, MUC4 and MUC16). Globular proteins also associate with the primary gel-forming mucin macromolecules MUC5AC and MUC5B, in what has been labeled the “mucin interactome”^29^. Held together largely by electrostatic and weak hydrophobic interactions, the mucin interactome contains ~ 30% of total globular protein content in healthy hydrated human ASL^29^. This sequestration changes with evolution of mucin concentration and ionic environment, altering mucosal viscoelastic, antimicrobial, and antioxidant immune function to degrees that have yet to be determined^29^. Evolution of globular protein sequestration following mucin concentration changes appears to occur gradually, over time scales ~ 30 min based on measured mucin/globular protein absorption and desorption times^29^, while relatively instantaneous with alterations in local ionic environment, such as abrupt changes in pH.

Dehydrated airways can be rehydrated by the inhalation of hyperosmolar aerosols^30^, as is commonly practiced in the treatment of cystic fibrosis (CF)^31^. Hypertonic aerosols such as hypertonic saline (HS) and mannitol^32^ increase rates of mucociliary clearance (MCC)^33^ by rehydrating the glycocalyx (periciliary layer) and mucus, thereby lifting mucus off compressed cilia for durations that can vary from ~ 30 min to several hours as a function of mucin, globular protein concentration, and sodium, chloride and water membrane permeabilities^34^. In hypersensitive airways, HS and mannitol can also trigger cough and bronchoconstriction^35,36^. Hyperosmolar aerosols with cell-permeating cation or lacking cell-permeating anion, such as NaCl and mannitol, provoke acute peaks of intra-cellular sodium and depressions of extracellular chloride^37^, inducing acute acidification^38,39^, and possibly triggering cough by TRPV1 activation^40^. Hypertonic divalent salts (HDS), such as endogenous MgCl_2_ and CaCl_2_, avoid acidification provocation for these same reasons^37^, hydrate for longer periods of time owing to the slow paracellular egress^37^ of the divalent cations Mg^++^ and Ca,^++^ and reduces cough events in hypersensitive airways^41^.

We wished to explore the mechanism of action and potential therapeutic value of HDS aerosols for rehydrating airways otherwise dehydrated airways on exposure to extreme atmospheres. We hypothesized that HDS aerosols with pH above the pKa of the cysteine disulfide linkages of MUC5AC and MUC5B macromolecules (8.3 in solution, while ranging from 7.4 to 9.1 in physiological environments)^42^ would partially and reversibly disassociate the mucin interactome^43^. We assumed this would liberate globular proteins to play their natural role as osmotic pumps, and thereby provide greater and prolonged hydration relative to HDS aerosols with relatively low pH. We further hypothesized that, by prolonging airway hydration, alkaline HDS rehydration would reduce compression for prolonged periods of time, and thereby enable a potentially practical and safe therapy for treating diseases of airway hypersensitivity provoked by the breathing of dry air.

We used continuum mechanics to analyze the response of airway mucosa to such high VPD levels before and after topical deposition of HS and HDS aerosols of variable pH. We used air liquid interface (ALI) cultures of human bronchial epithelial (HBE) cells to study gene expression associated with airway hypersensitivity on exposure to air with high VPD, and the rehydration behavior of the ALI cultures in response to topical deposition of HS and HDS aerosols of variable pH. We finally conducted a randomized, double blind, placebo-controlled study in 10 refractory chronic cough (RCC) patients (REACH) in the hot and dry climate of Riyadh, Saudi Arabia to evaluate the cough suppression treatment effect of HDS pH 9 aerosols, and compared these results with findings determined from a recent clinical trial in the moderate climate of London with 12 RCC patients treated by HDS pH 8 and pH 9 aerosols. The results of our studies are reported here.

## Results

### High alkalinity facilitates reversal of mucosal collapse caused by heatwave exposure

We evaluated by continuum mechanics the response of human upper airway epithelial cells following exposure to a heatwave atmosphere, and subsequently on exposure to hydrating aerosols of hypertonic (monovalent) salts (HS) and hypertonic divalent salts (HDS) of variable pH (Methods). As a base case, we considered an atmosphere of VPD ~ 1.5 kPa, typical of mouth breathing in a dry indoor atmosphere. Chronic mouth breathing, recently estimated to afflict 40–50% of children^44^ and adolescents^45^, induces a base-case average evaporation rate *q* sufficient to thin airway mucus from the larynx to bronchiole generation 6 (Supplemental Information)^10^. Mucosal thinning accompanies collapse onto cilia within the trachea, primary, secondary and tertiary bronchi in this same base case (Fig. 3A). Mucosal collapse compresses the dehydrated airway epithelial cells by an amount (Methods)

1 $$\Delta P_{d} = \frac{q}{{L_{p} }} - K_{PCL - d} \Delta \Pi_{gp - 0} - \Delta \Pi_{tm - 0} $$

with *L*_*p*_ the hydraulic permeability of the apical epithelial cell membrane, ΔΠ_*gp*-0_ and ΔΠ_*tm*-0_ the osmotic pressures of globular protein (~ 350 Pa) and tethered mucin (~ 180 Pa) in the fully hydrated “0” state, and *K*_*PCL-d*_ the partition coefficient of globular protein from the dehydrated mucus into the PCL. In the base case scenario, it follows that, on average within the larynx, trachea and through the 5th airway bronchiole airway (Supplemental Information), VPD ~ 1.5 kPa, *K*_*PCL-d*_ = 0.44, while at the heatwave extreme condition (Fig. 1C) of atmospheric VPD = 6 kPa, average airway VPD ~ 2.7 kPa in these same airways, *K*_*PCL-d*_ = 0.19. Mechanical compression of upper-airway epithelial cells follows from Eq. (1) as Δ*P* = 0.98 kPa for the base case and Δ*P* = 2.68 kPa for the heatwave case, near the ~ 3 kPa acute kPa threshold that has been implicated in the provocation of cough^25^ and bronchospasm^26^.

**Fig. 2:**
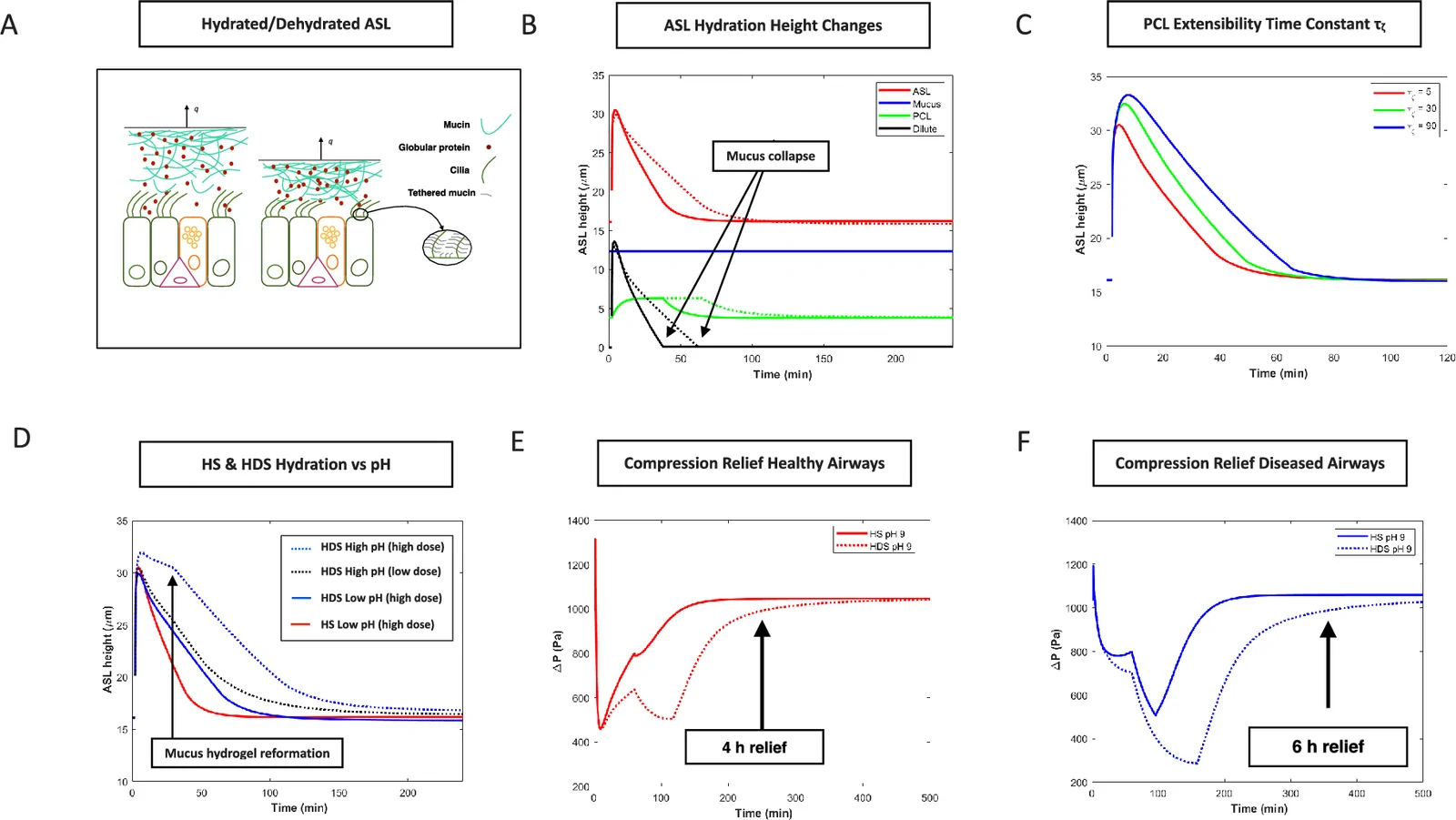
Numerical simulations of human airway rehydration post exposure to aridity. Rehydration alters ASL structure and alleviates the compression of airway epithelial cells. (**A**) Cross section of a healthy ASL above a human airway epithelium comprising ciliated epithelial cells, goblet cells and basal progenitor cells. Mucin tethered to cilia and the apical epithelial membrane and globular protein provide the principal osmotic pressure of the ASL in steady state. On exposure to warm humid air, characteristic of slow nasal breathing, the ASL is fully hydrated. On exposure to arid conditions, the mucus layer thins and collapses on cilia. (**B**) Change in the heights of ASL, mucus hydrogel, dilute mucin layer, and PCL vs time post deposition of HS (NaCl, 4.3% w/w, pH 7) (solid lines) and HDS (MgCl_2_, 4.3% w/w, pH 7) (dashed lines) with total deposited mass 25% of ASL mass. Airway generations 0 to 6 in normal tidal breathing (15 L/min), or generations 0 to 9 with fast breathing (30 L/min), are assumed dehydrated by the mouth breathing of 25 oC, 30% RH air, resulting in exposure to an average VPD ~ 1.5 kPa (ranging from ~ 3.4 kPa in the larynx to ~ 0.5 kPa in the first bronchioles). (**C**) Change in the height of ASL following deposition of HS (NaCl, 4.3% w/w, pH 7) versus cilia extensibility time *τ*_*ζ*_. (**D**) ASL height post deposition of HS and HDS aerosols at low (*K*_*PCL*-0_ = 0.7) and high pH (*K*_*PCL*-0_ = 3.0). (**E**) Epithelial cell compression post alkaline HDS (MgCl_2_, 4.3% w/w, pH 9) deposition versus HS (NaCl, 4.3% w/w, pH 9) for “healthy airways” with fast cilia extensibility (*τζ* = 5 min) and normal mucus thickness (*h*0 = 20 μm). (**F**) Epithelial cell compression post alkaline HDS (MgCl_2_, 4.3% w/w, pH 9) deposition versus HS (NaCl, 4.3% w/w, pH 9) for “diseased airways” with slow cilia extensibility (*τ*_*ζ*_ = 30 min) and hyper-secreted mucus thickness (*h*_0_ = 40 μm). Source code available on request. 1

We examined numerically the consequence of rehydrating dehydrated human airways by topical deposition of non-alkaline HS (NaCl) and HDS (MgCl_2_) aerosols (Fig. 2B). Following deposition, a dilute mucin layer immediately grows above the PCL (black lines), lifting dehydrated mucus off compressed cilia, allowing the PCL (green lines) to expand on an extensibility time scale *τ*_*ζ*_ assumed ~ 5 min. Water uptake is negligible in the mucus layer given the relatively rapid diffusion of the least mobile constituents of the ASL (*l*^2^/*D* ~ 10^–12^ m^2^/10^–12^ m^2^/s ~ 1 s) in comparison to mucus swelling time (minutes). Continued evaporation therefore retains the transpiring hydrogel mucin stratification and mucus thickness (blue line). Slow clearance of the divalent cation relative to the monovalent cation prolongs hydration for HDS (dashed lines). The speed of dehydration post-topical deposition of HS or HDS aerosols depends on the extensibility time *τ*_*ζ*_ (Fig. 2C), or severity of airway dehydration. This prolongation of hydration in severely dehydrated or diseased airways (*τ*_*ζ*_ >  > 5 min) is owed to the greater osmotic pressure of the compressed PCL, resulting in greater water efflux into the ASL.

Duration of hydration is further extended, at all extensibility time scales, with deposition of high pH HDS (Fig. 2C). For large deposited masses (25% ASL mass), ASL pH increase is significant, rising from ~ 7 to ~ 7.5 in the case of ASL of neutral pH. This rise in ASL pH, given the relatively acidic nano-environment of the glycocalyx^46^, is assumed to increase partitioning (*K*_*PCL*-0_) of globular protein into the PCL, increasing osmotic water flow into the ASL (Fig. 2D). For deposited masses less than 10% ASL mass, ASL pH rise is negligible, and the consequence of globular protein liberation from the disordered mucin interactome is a relatively modest increase in the globular protein osmotic pressure (Fig. 2D). Airway epithelial cell compression falls post deposition (Fig. 2E, F) owing to globular protein release from the disrupted mucus hydrogel. The spike in compression on deposition in Fig. 2E, F reflects the assumed stepwise change in *K*_*PCL*-0_ from 0.7 to 3. A gradual increase of *K*_*PCL*-0_ over the first minutes of hydration, the more likely consequence of the natural process of mucin interactome disentanglement, combined with the rapid ASL volume growth and dilution of globular protein concentration, should smooth out the compression response, favoring a more gradual diminution of compression over the first minutes of rehydration, as globular protein accumulates in the PCL post breakup of the mucin interactome.

**Fig. 3:**
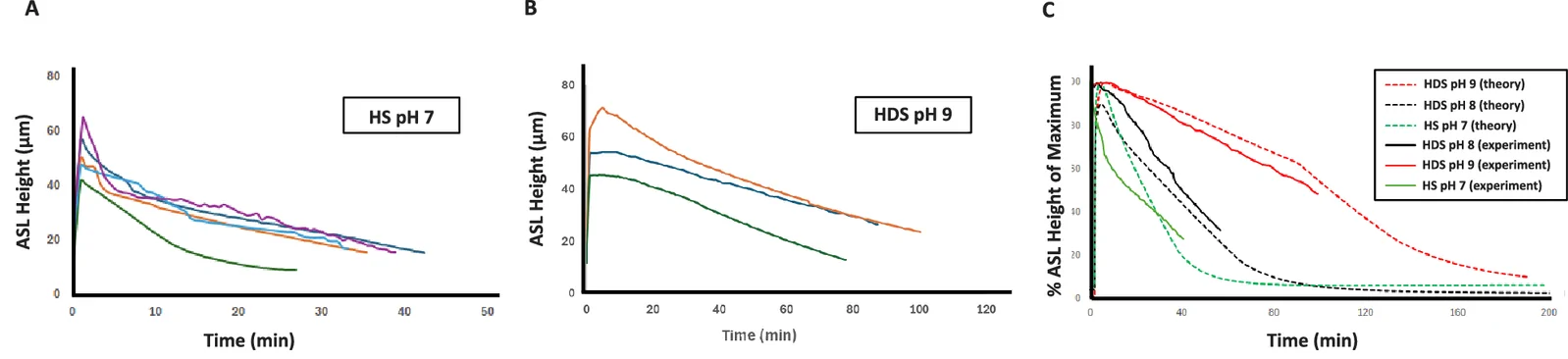
Experimental findings following rehydration of ALI cultures of HBE cells versus salt type and alkalinity. ASL height versus time following nebulization of (**A**) HS (4.3% w/w NaCl) (n = 5) and HDS (4.3% w/w MgCl2) pH 9 (n = 3) (**B**) measured at 6 lateral points in the plane of the confocal microscope lens. (**C**) ASL height normalized by peak ASL height averages following nebulization of HS (4.3% w/w NaCl) (n = 5) and HDS (4.3% w/w MgCl_2_) pH 8 (n = 3) and pH 9 (n = 3). Theoretical predictions are based on the model parameters of Fig. 2 with *τ*_*ζ*_ = 5 min and mucus reformation time (*T*_*M*_) = 90 min. Source data provided here. 1

We explored whether rehydration of dehydrated ASL by hypertonic salts would follow the predicted behavior shown in Fig. 2. We therefore exposed ALI cultures of HBE cells to a moderately arid heatwave atmosphere of 35°C, 60% RH (VPD = 2.2 kPa) (Fig. 1C). We exposed the ALI cultures to this atmosphere for 1 h in a humidity-controlled confocal microscope chamber with air circulation. We then nebulized onto the dehydrated ASL HS (4.3% w/w NaCl) pH 7 and HDS (4.3% w/w MgCl_2_) pH 8 and 9. We monitored ASL height over time as described in the Methods and our results are shown in Fig. 3A and B. Since mucus height varied from sample to sample, we normalized each set of data by the maximum ASL height achieved and compared the normalized data as shown in Fig. 3C. Also shown are the theoretical predictions based on a PCL extensibility time scale *τ*_*ζ*_ = 5 min, consistent with the acute nature of the dehydration of healthy epithelial cells.

### Heatwave exposure up-regulates genes associated with cough hypersensitivity

We assessed the impact of prolonged mucosal collapse by arid atmosphere exposure on the expression of genes associated with cough hypersensitivity by exposing ALI cultures of HBE cells to a heatwave atmosphere (Fig. 1C) of 35 ºC air with 30% RH (VPD = 4.4 kPa) for 8 h in a humidity-controlled confocal microscope chamber with air circulation (Methods). As control, we exposed ALI cultures of HBE cells to a highly humidified atmosphere of 35 ºC, 95% RH (VPD = 0.5 kPa). After 3 h of exposure at the arid heatwave (35 ºC, 30% RH) condition, and at both the 30% and 95% RH conditions after 8 h of exposure, cells were withdrawn, RNA extracted, and bulk RNA-sequence data analyzed computationally (see “Methods”). We assessed the following genes associated with sodium, chloride and water regulation or otherwise implicated in cough hypersensitivity—ANO1, AQP1, AQP5, CFTR, F2R1, ORAl1, P2RX3, P2RX4, P2RX7, P2RY2, PIEZO1, PIEZO2, SCNN1A, SCNN1B, SCNN1D, SCNN1G, TRPA1, TRPV1, TRPV2, TRPV3, TRPV4, TRPV5, TRPV6 (Supplemental Information).

**Fig. 4:**
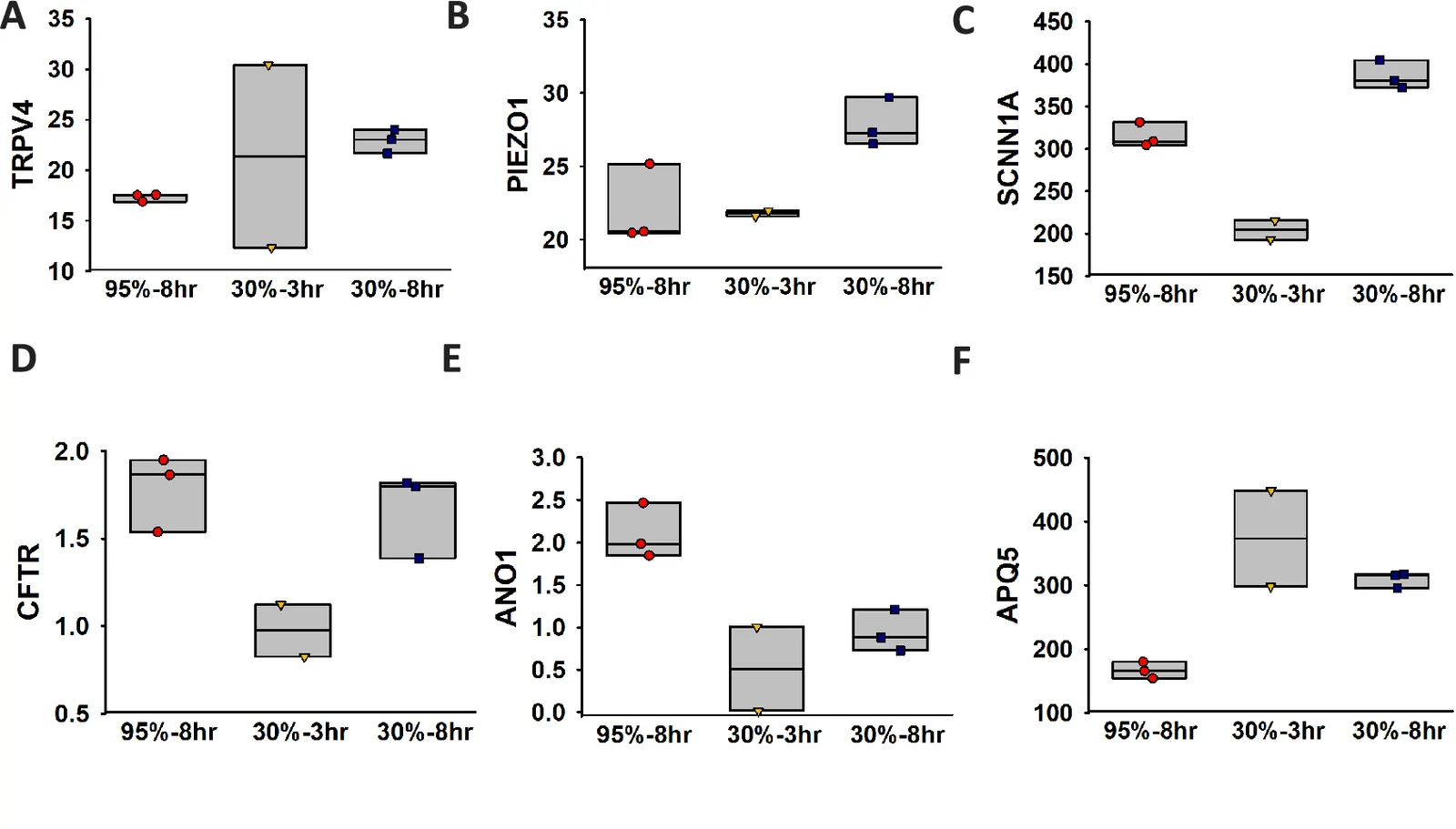
Dehydration and rehydration of ALI cultures of HBE cells. (**A**–**C**) Bulk RNA-seq gene expression results for (**A**) TRPV4, (**B**) PIEZO1, (**C**) SCNN1A, (**D**) CFTR, (**E**) ANO1, (**F**) AQP5 following exposure of ALI cultures of HBE cells to air at 37 °C and 95% RH and 30% RH (n = 3) for 8 h.

Figure 4A–F highlight results for six MSC genes particularly critical to peripheral cough hypersensitivity. Gene expression of TRPV4 (p = 0.009) and PIEZO1 (p = 0.0432) rises significantly at 8h at the high VPD (heatwave) condition compared to the low VPD control (Fig. 4A, B), consistent with reported TRPV4 and PIEZO1 up-regulation following mechanical compression of epithelial cells.^47,48^ No significant change in either gene is observed at 3 h of exposure (p > 0.05), suggesting no up-regulation of cough hypersensitivity in the first hours post dehydration. Gene expression of the α subunit ENaC protein SCNN1A (Fig. 4C) falls after 3h at 30% RH (p = 0.0105) relative to 95% RH, while after 8h aridity exposure SCNN1A expression rises (p = 0.0057) relative to the humid condition. A similar trend is observed for the β subunit of ENaC (Supplemental Information). Down-regulation of ENaC appears to be a natural defense against dehydration, and particularly against down-regulated CFTR,^49^ as occurs (Fig. 4D) at 3h of exposure (p = 0.0439). ENaC expression rises from 3 to 8h consistent with reports of up-regulation of ENaC under mechanical stress,^18^ suggesting that the immune defense of the airways to dehydration is surpassed with prolonged dehydration > 3h. CFTR expression tends to down-regulate (Fig. 4D), reflecting CFTR down-regulation on prolonged ATP exposure^50^ consequent to TRPV4 compression enhancing hypersensitivity by airway dehydration and acidification. TRPV4-mediated ATP secretion further triggers cough hypersensitivity by stimulating P2X3 receptors, which also up-regulate at 8 h of dry air exposure while not at 3 h of exposure (Supplemental Information).^51^ Expression behavior of the ANO1 chloride channel gene (Fig. 4E) parallels the behavior of CFTR (Fig. 4D), whereas the up-regulation at 8 h of aridity exposure (p = 0.0001) of AQP5 gene, encoding one of the two principal water channels in airway epithelial cells, appears to be a direct response to the dehydrated state (Fig. 4F).

### Alkaline HDS aerosols diminish cough hypersensitivity in RCC patients

We hypothesized that mucosal collapse in the vicinity of the larynx might play a role in the development of cough hypersensitivity, such that inhalation of alkaline HDS aerosols in RCC might provide treatment relief of daily cough bout counts, and that daily cough bout counts might remain suppressed post-treatment. We therefore conducted a randomized, double blind, placebo controlled study (REACH) in 10 RCC patients in Riyadh, Saudi Arabia (Methods), where monthly VPD ranged from 1.51 to 7.25 kPa, typical of heatwave day extremes, which single-model climate analysis suggests will characterize the majority of summer days in urban centers of the United States by the last 40 years of this century (Supplemental Information). We evaluated the placebo-adjusted efficacy of cough and cough bout rate suppression following two weeks treatment with an oral inhaled HDS pH 9 aerosol relative to a normal saline orally inhaled placebo control. We further evaluated cough and cough bout counts for several weeks post treatment. We defined cough bouts^52^ as 2 or more consecutive coughs with 2 s or less interval and monitored continuously using a Hyfe digital cough watch monitor^53^ We compared the results of the REACH study with the results of a recent clinical trial^54^ in 12 RCC patients following one week treatment with HDS pH 8 and pH 9 aerosols. In both trials patients self-administered the aerosol 4 times per day and used an identical inhaler for the active and placebo administration.

**Fig. 5:**
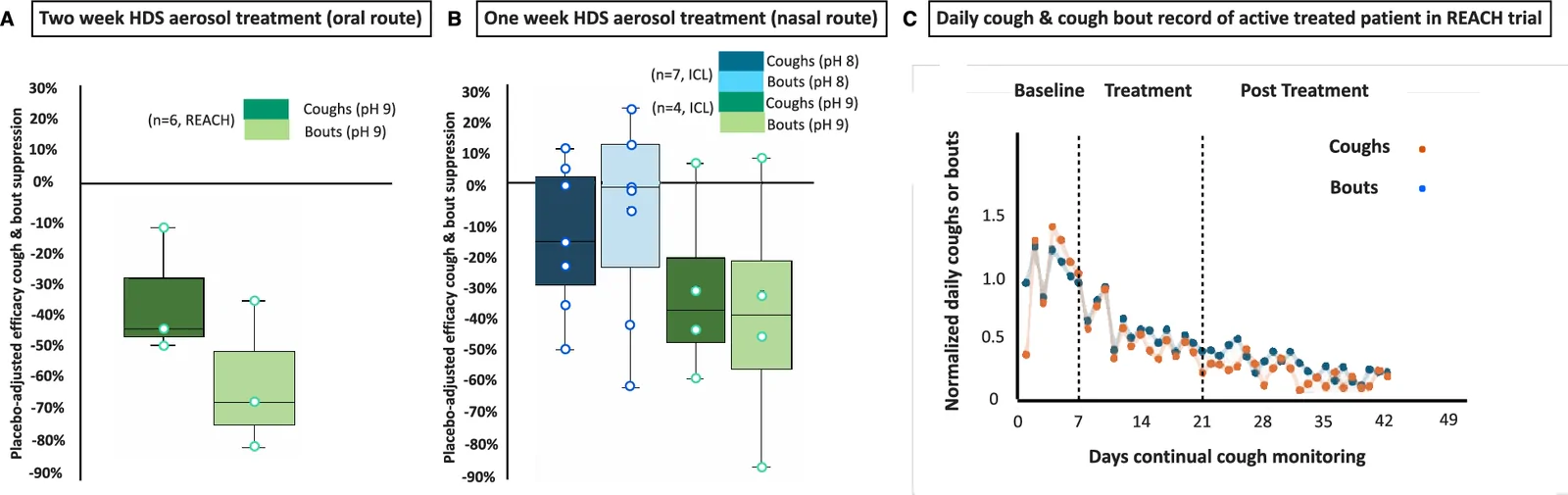
Suppression of cough and cough bouts in the REACH trial. (**A**) Placebo-adjusted efficacy of daily cough and cough bout rate in the REACH trial for 6 RCC patients (3 active and 3 control) after two weeks of treatment with HDS pH 9 with efficacy based on average of cough and cough bout rates over the last 4 days of the treatment period. (**B**) Placebo-adjusted efficacy of daily cough and cough bout rate for 12 RCC patients in the ICL trial^58^ (7 treated with HDS pH 8, and 5 with HDS pH 9) after one week of treatment with efficacy based on average of cough and cough bout rates over the last 3 days of the treatment period. (**C**) Daily cough and cough bout rates normalized by baseline values for subject C (with baseline cough rate 24.5 coughs/hour, see Supplemental Information) versus time during 7 day baseline, 2-week treatment, and 3 weeks post treatment. Source data provided here.

Relative to placebo, cough bout frequency diminished by 59% (p = 0.03) for those RCC subjects treated by HDS pH 9 aerosol, and cough rate frequency by 34% (p = 0.045) after two weeks daily oral inhalation (n = 6) (Fig. 5A). This compared to 41% (p = 0.03) and 35% (p = 0.03) for cough bout and cough frequency suppression with HDS pH 9 from the previous study^53^ after one week of nasal administration in RCC patients (n = 4), and 16% (p = 0.03) and 4% (p > 0.05) with HDS pH 8 aerosol (n = 7)(Fig. 5B), consistent with the prolonged hydration achieved by the pH 9 divalent salt aerosol relative to pH 8 (Fig. 3C), providing prolonged airway epithelium compression relief (Fig. 2F) Ratio of average daily baseline coughs to cough bouts in the REACH trial was 5.0 ± 1.2 for all (9 or 10) intention-to-treat (ITT) patients with daily cough rate of 5 coughs/hour or greater (Methods). This ratio increased to 8.0 ± 2.8 (n = 3) on HDS pH 9 treatment, while it diminished to 4.4 ± 0.4 (n = 3) on placebo treatment, suggesting the need for a greater number of coughs to provoke a cough bout, or an apparent reduction in the hypersensitivity of the airway epithelium.

Daily cough bouts, and daily coughs, remained suppressed post treatment in the REACH trial, as illustrated in the daily cough record of the active-treated patient shown in Fig. 5C. For the entire group (see Supplemental Information for the daily cough and cough bout record of all six active and placebo patients), cough bout frequency remained suppressed relative to placebo by 48% one week post treatment (p = 0.01) (n = 6), a further indication of loss of cough hypersensitivity following treatment of the upper airways with the HDS pH 9 aerosol.

## Discussion

Heat waves elevate incidence of cough and bronchospasm, increase hospitalization rates of asthmatics and sufferers of COPD^2^, and threaten the respiratory health of vulnerable populations, including children^55^, the elderly^56^, and low-income populations^57^. This respiratory distress is likely to dramatically increase in the coming decades, with unprecedented heatwave burden estimated to afflict between 52 and 92% of humans born today by the end of this century based on a 1.5 to 3.5°C rise in global temperatures^1^.

Within this rising heatwave burden, rising burden of atmospheric aridity, or VPD, appears to be a particularly serious threat to respiratory health. We find that exposing human bronchial epithelial cells to an atmosphere within the VPD range characteristic of the June 2026 European heatwave causes mucosal collapse, compression of airway epithelial cells, and up-regulates TRPV4 (Fig. 4A), PIEZO1 (Fig. 4B) and SCNN1A (Fig. 4C) following 8 h of exposure, while not following 3 h of exposure. Up-regulation of these genes increases the likelihood that relatively small mechanical provocations result in relatively large secretions of ATP (TRPV4)^19^, inflammatory cytokines (TRPV4, PIEZO1)^16,17,19^ and airway acidity (SCNN1A)^18^ thereby increasing the likelihood of airway hypersensitivity on the breathing of hot atmospheres.

Relief from the inflammatory compression accompanying mucosal collapse appears to occur for less than 3 h on the inhalation of non-alkaline HS and HDS aerosols (pH < 8), while greater than 3 h following the inhalation of alkaline HDS aerosols with pH > 8 (Fig. 3C), a rehydration effect that is predicted to increase in diseased airways (Fig. 2E vs F) owing to greater retention of water in the more viscous diseased mucus. We attribute this prolonged hydration to the reversible breakup of the mucin interactome at high pH, combined with slow clearance of the divalent cation (Mg^++^ or Ca^++^). Similarly prolonged hydration has been observed in ALI cultures of HBE cells following topical deposition of hypertonic non-alkaline HS aerosols via blockage of ENaC or on stimulation of AQP5^34^. Within the pH excursion post deposition of the HDS pH 9 aerosol (~ + 0.5), ENaC permeability appears to diminish by ~ 15%^58^, insufficient to account for the prolonged hydration observed, while AQP5 permeability is unchanged^59^. Relatively long hydration times have also been observed to occur in highly concentrated CF-like ALI cultures, and attributed to the elevated (globular-protein-dominated) osmotic pressure inherent to highly concentrated ALI cultures of HBE cells^60^.

Pig gastric mucus has been reported to dissolve at pH above 8.5, near the pKa of cysteine that forms the primary amino acid linkages between MUC5AC and MUC5B macromolecules in mucus hydrogels^43^. Our continuum mechanic analysis (Methods) predicts that, by such a phase transition in the vicinity of the deposited aerosol, globular proteins are freed from the mucin interactome to function as freely-diffusing osmotic pumps (Fig. 2D). While globular protein concentrations in healthy ASL are far lower than those observed in CF-like airway mucosa, deposition of the alkaline hypertonic aerosol appears to lead to an accumulation of globular protein near the airway epithelium, achieving local concentrations similar to the hyper-secreted mucin ASL of CF-like airways. We postulate that this accumulation owes to temporary elevation of ASL pH relative to the acidic environment maintained in the vicinity of the glycocalyx. Osmotic water flow into the ASL therefore increases to the degree experimentally observed (Fig. 3C), sustaining compressive stress release for approximately 4 to 6 h depending on the degree of dehydration (Fig. 2 E, F).

The persistency of cough and cough bout suppression observed in the REACH trial post treatment (Fig. 5C) is consistent with our theoretical (Fig. 2) and experimental (Fig. 3) findings that MSC-related airway hypersensitivity is dependent on airway hydration state. Particularly, prolonged hydration appears to reduce compression of the laryngeal/tracheal epithelium, and with this reduction secretion of cough triggers diminish. Over many days of treatment, a loss of hypersensitivity appears to explain the reduced tendency to cough post treatment. This interpretation may further explain the increase in the cough/bout ratio observed following treatment by the HDS pH 9 aerosol, as is reflected in the greater mean reduction in cough bouts relative to coughs (Fig. 5A, 5B).

Many avenues of research remain to be explored. Mucin interactome evolution in extreme atmospheres likely impacts airway immune function in multiple ways, ranging from MSC activation to anti-microbial function. Molecular, cellular, bacterial and viral interactions and consequences to the pathogenesis of respiratory disease should all be investigated. Alkaline endogenous ion treatments for other diseases of the airways, including asthma, allergic rhinitis, and bronchiectasis should be explored, as well as non-endogenous compositions that might improve on the outcomes of endogenous therapies, possibly providing longer action and more profound relief from the compressive inflammatory state. The potential prophylactic benefit of managing airway hydration during heat waves by the daily inhalation of non-alkaline and alkaline endogenous ion aerosols should also be investigated, as well as acute relief treatments following wildfire or burn-pit exposure.

Heat wave impact on airway hypersensitivity mirrors heat-wave impact on crop failure rate^1^. Topical hydration will be necessary to avoid the 3–4 fold increase in crop failures predicted to occur with current climate forecasts over the course of this century^1^. For similar mechanistic reasons, airway hydration management and treatment strategies may be critical to avoid a crisis in human respiratory illness.

## Methods

*Human bronchial epithelial cultures* Non-diseased airway epithelial cells were obtained from deceased donors under the auspices of the University of North Carolina Institutional Review Board (IRB). Epithelial cells were isolated from the large airways using previously published methods. Isolated primary cells were grown to ~ 80% confluence in collagen-coated flasks with the appropriate growth media^61^ prior to being passaged onto collagen-coated 1 cm^2^ supports (Costar Transwell; 3 µm pore size) at a density of ~ 1.25 × 10^5^ cells/cm^2^. The cells were cultured at air–liquid interface and maintained at 37 °C, 5% CO_2_, and 95% relative humidity (RH) until fully differentiated (21–28 days). During culturing, accumulated mucus was removed by PBS lavage once per week.

*Bulk RNA-seq gene expression analysis* The media of well-differentiated human airway epithelial cultures was replaced at T = 0 before being placed in incubators that were maintained at either 95% or 30% humidity (as described for the ASL height studies). All cultures had 3 days of accumulated mucus to simulate non-diseased concentration. After 3 h of exposure (for 30% humidity) or 8 h (at 30% and 95% humidity), cells were quickly washed with PBS (1 min) before QIAzol (Qiagen) was added to lyse cells. Lysed cells were kept at − 80 ºC until ready for subsequent processing. Total RNA from each Transwell was extracted using a Total RNA extraction kit (Qiagen) and submitted to Novogene (Sacramento, CA) for mRNA purification, cDNA library construction, sequencing (using a Novaseq 6000 platform), and resulting data quality checking.

*Computational analysis for bulk RNA-seq data* Sequence reads in FASTQ format, obtained from Novogene, were aligned to the GRCh38 reference genome with Gencode v46 annotations for exons and splice sites using the STAR aligner^61^. Transcript abundance was quantified as raw counts and transcripts per million (TPM) using StringTie assembler^62^. For normalization, raw counts were processed with the voom function from the Bioconductor limma package, employing mean–variance weighing on log-cpm values^63^. Differential expression was analyzed through linear models using the Bioconductor package, *limma*^64^.

*REACH trial* A double-blind, randomized, placebo controlled crossover study (NCT07003347, registered on ClinicalTrials.gov on May 25, 2025) at King Saud University (KSU) was conducted to examine the safety and efficacy of SC0023, an alkaline HDS with pH 9, on cough suppression in 10 intention-to-treat (ITT) RCC participants administered by oral inhalation against placebo (oral saline aerosol). The study was conducted in accordance with the recommendations for physicians involved in research on human subjects adopted by the 18th World Medical Assembly, Helsinki 1964 and later revisions. The protocol was approved under medical device regulations by the Saudi Food and Drug Administration (SFDA), and the protocol, patient-informed consent form, and patient recruitment materials were reviewed and approved by and the Research Ethics Committee (REC) of King Saud University. Participants signed patient-informed consent forms before enrolling in the study. This consent ensured the right of the participant to refuse to participate without giving reasons, and to withdraw at any time from the protocol treatment without giving reasons and without prejudicing further treatment. Both SC0023 and placebo were delivered with the same handheld pump-spray. Coughs were measured using a HYFE digital cough monitor. Participants diagnosed with RCC for at least 6 months and a visual analogue score (VAS) > 40 mm were enrolled into the study. Exclusion criteria included respiratory tract infection within the last 3 weeks, a concomitant diagnosis of underlying airways disease or vocal cord disorder, a forced expiratory volume in 1 s (FEV1) < 70% predicted, a history of current smoking/ex-smokers with > 10 pack- year history and long-term use of angiotensin-converting-enzyme (ACE) inhibitors and immunosuppressive treatments. Of the fifteen participants screened, and 10 patients enrolled onto the study (Table 1), 3 of the participants had protocol violations, and post-unblinding, one of the patients was determined to have received less than a single dose of the active during his active treatment period and was therefore excluded in the post-hoc analysis. In this randomized, double-blinded, placebo-controlled crossover study, participants attended a total of 5 visits over a 12- to 14-week period. At screening (visit 1), enrolled participants were provided with a digital cough watch monitor to record continuous hourly cough counts during baseline assessment (Week 1), during a first treatment period of two weeks during which they were randomly assigned to groups A (active treatment) or B (placebo treatment) (Weeks 2 and 3), during a washout period of 3–5 weeks (Weeks 4 to 7 and possibly up to 9), during a second baseline week (Weeks 8, 9 or 10) and during a second treatment period of two weeks where patients in the A and B groups during the first treatment period crossed over to the B and A groups (Weeks 11–12, 12–13 or 13–14). Participants were provided with training to ensure correct use of both the digital cough watch monitor and soft mist spray. Participants were blinded as to whether they received active or control. Upon unblinding of the study, carryover effect was assessed, reflecting the prolonged treatment effect of SC0023. Given the observation of a significant treatment period effect primary efficacy analysis of relative change from baseline in 24-h cough frequency the analysis was limited to the first dosing period as a parallel arm design. Post hoc analyses of the data considering adherence of the investigational product showed a 34% (n = 6) placebo adjusted reduction in cough rate (p = 0.045), similar to the findings in a previous clinical trial with SC001^54^. Visual analog score (VAS) was observed to fall by 40 relative to placebo (n = 6) and similarly across baseline cough rates—falling 56 for baseline cough rate of 5 coughs/hour and falling 54 for baseline cough rate of 25 coughs/hour. Persistency of cough rate suppression, as monitored using the Hyfe cough monitor, continued for several weeks post dosing notwithstanding the 3-h clearance time of SC0023 endogenous ions as is consistent with treatment de-compression and down-regulation of mechano-sensitive ion channels (MSCs) implicated in cough hypersensitivity. There were no clinically significant treatment emergent adverse events reported, and two SAEs reported were deemed not related to the investigational product. The raw daily cough counts for each of the six subjects in the post-hoc group are shown in Fig S6.

**Table 1:** Participants’ demographics, anthropometrics, and vital signs (N = 10).

Variable	Active	Placebo
Age (years)	42.4 ± 17.1	51.2 ± 14.5
Mean ± SD		
Median (Q1–Q3)	45 (26–57.5)	58 (37–62)
Min–Max	23–66	29–66
Sex, n (%)	1 (20.0%)	3 (60.0%)
Female		
Male	4 (80.0%)	2 (40.0%)
BMI* (Kg/m^2^)	31.5 ± 9.6	26.8 ± 7.9
Mean ± SD		
Median (Q1–Q3)	27.8 (26.4–38.5)	28.6 (19–33.8)
Min–Max	24.9–48.5	16.8–36.8

*BMI: body mass index.

*Continuum mechanics model background* Consider a healthy, confluent, human epithelial cell layer in the presence of an atmosphere of fully saturated air at or near body temperature. Above the epithelial cell layer is an airway surface liquid (ASL) layer comprising a periciliary layer (PCL) and mucin-rich mucus layer. The fully hydrated PCL is of height *l*_*PCL-*0_ = 7 μm, comprising elastically deformable cilia, with a cilia-tethered and cell-surface-tethered mucin brush, possessing osmotic pressure Π_*tm*-0_ ~ 180 Pa^27^. The mucus layer is of height *h*_0_ = 20 μm and rich in soluble mucin strands (0.5% by weight). Distributed throughout the ASL are 0.9% salts and 1.1% globular proteins. In time average over many breaths, salt ions distribute between the ASL and surrounding epithelial cells and tissue, such that the very large osmotic pressure of the salt ions (~ 10^5^ Pa) balances across surrounding membranes and tissues, while globular proteins, which highly associate with mucin macromolecules through electrostatic, hydrophobic and covalent interactions, maintain a significant ASL osmotic pressure Π_*gp*-0_ ~ 350 Pa^27^. On exposure to unsaturated air of some relative humidity (RH) and temperature *T* characteristic of inhaled air, water evaporates from the free surface at a rate *q*, which grows with increasing *T* and diminishing RH*,* reflecting a proportional dependence on vapor pressure deficit, VPD, and related by the Penman-Shuttleworth equation (Supplemental Information)

$$q=\frac{1.83\left[1+0.536\left(\frac{2}{15}{V}_{R}\right)\right]}{m+0.054}\times \Delta \times {10}^{-8}$$

Here, *q*(m/s), *V*_*R*_ is the ventilation rate (L/min), *m* the slope of the saturation pressure (kPa/C) versus temperature (^o^C) curve at the particular temperature *T*, and Δ is the VPD (kPa) in the particular airway region. Water evaporation alters the mucin structure of the ASL over time scales larger than the oscillatory time scale of many breaths (*q*^2^/*D* with *D* the diffusivity of mucin strands), while shorter than the time scale of evolution of the mucin-associated structure of the ASL. This evolution occurs through a process of mucus transpiration^10^ that scales in intensity with increasing rates of evaporation relative to mucin diffusion, as represented by a dimensionless Péclet number, *Pe*_0_ = *qh*_*0*_/*D*. Qualitatively, mucus transpiration occurs as follows:^10^ Water moves across the ASL in the steady state at a rate *q* to maintain ASL volume, driven by osmotic imbalances across the apical epithelial membrane. This convection drags mucin macromolecules toward the lumen, establishing a mucin concentration gradient over the thickness of the mucus layer, which grows with increasing evaporation rate *q*. At a critical height above the PCL (Fig. 1B), mucin mass fraction reaches its overlap mass fraction 0.006 and reversibly gels. Water now moves through the mucus hydrogel, of thickness *l*_*m*_, at the rate *q* by the action of the mucin osmotic pressure gradient, while, beneath the hydrogel, in the dilute mucin layer, of thickness *l*_*dil*_, and in the PCL, water moves at velocity *u* under the total osmotic pressure of Π_*PCL*_ = Π_*gp*-0_ + Π_*tm*-0_ across the airway epithelium. The mucin osmotic pressure gradient grows with increasing evaporation rate owing to increasing mucin concentration near the evaporating surface. Also contributing to the osmotic pressure gradient is thinning of the mucus hydrogel, which occurs by mucin mass conservation^10^, noting that mucin size precludes penetration into the PCL. The elevated osmotic pressure gradient maintains the movement of water through the hydrogel at the rate *q*. At sufficiently high evaporation rate, osmotic water movement across the apical epithelial membrane falls below the value of *q*, such that the dilute layer vanishes, and the mucus hydrogel collapses onto the PCL, pressing the latter until the osmotic pressure in the PCL rises to generate an osmotic velocity equal to the evaporation rate and to conserve the dehydrated ASL volume in the new steady state.

*ASL heights after dehydration* In this dehydrated state, the height of the ASL

2 $$H\left(t\right)={l}_{m}\left(t\right)+{l}_{PCL}\left(t\right)+{l}_{dil}\left(t\right)$$

assumes a steady-state value *H* = *H*_*d*_*,* where *l*_*m*_ = *l*_*m-d*_, *l*_*PCL*_ = *l*_*PCL-d*_, *l*_*dil*_ = *l*_*dil-d*_ = 0, or

3 $${H}_{d}={l}_{m-d}+{l}_{PCL-d}$$

Here, the dehydrated mucus height is given by^10^

4 $$\frac{{l}_{m-d}}{{h}_{0}}=\frac{3}{{Pe}_{0}}\left[{\left(1+\frac{4}{3}{Pe}_{0}\right)}^{1/4}-1\right]$$

and the dehydrated PCL height by the approximation (Supplemental Information)^27^

5 $${l}_{PCL-d}\approx {\zeta}_{d}+\frac{{\zeta}_{d}^{2}}{10}$$

where, in the fully hydrated state, the correlation length *ζ* = *ζ*_0_ = 4.4 μm, representing the mesh size between tethered mucin in the PCL. The dehydrated correlation length (${\zeta}_{d}$) can be approximated in terms of the osmotic pressure of the tethered mucin in the dehydrated layer (${\Pi}_{tm-d}$) by^27^

6 $${\zeta}_{d}\approx {l}_{PCL-0}\left[1-exp\left({\left(-\frac{180}{{\Pi}_{tm-d}}\right)}^{1/3}\right)\right]$$

with (Supplemental Information)

7 $${\Pi}_{tm-d}=\frac{q}{{L}_{p}}-\frac{{K}_{PCL-d}{\Pi}_{gp-0}}{{\left(1+\frac{4}{3}\frac{{\phi }^{*}}{{\phi}_{c}}\right)}^{1/4}-1}$$

Here, *L*_*p*_ (~ 0.8 × 10^–10^ m^2^-s/kg)^10^ is the hydraulic water permeability of the apical epithelial membrane (~ 0.8 × 10^–4^ m/s = *L*_*p*_*R*_*g*_*T*_*k*_/*v*_*w*_ where *v*_*w*_ = 18.14 × 10^–6^ m^3^/mol is the molar of water, *R*_*g*_ = 8.314 J/^o^K-mol is the gas constant and *T*_*k*_ = 310^o^K). Π_*gp*-0_ ~ 350 Pa is the fully hydrated state osmotic pressure of globular protein, ${\phi }^{*}=0.005$ the fully hydrated mass fraction of mucin and ${\phi }^{c}=0.006$ the mucin overlap mass fraction. The partition coefficient *K*_*PCL*_ of globular protein into the PCL, with value^15^ *K*_*PCL*-0_ = 0.7 in the fully hydrated state, is assumed to obey

8 $${K}_{PCL}={K}_{PCL-0}\left[\frac{{l}_{m}}{{h}_{0}}+\left(\frac{{l}_{m}}{{h}_{0}}-\frac{{\phi }^{*}}{{\phi}_{c}}\right)\right]exp\left(\frac{{l}_{m}}{{h}_{0}}-\frac{{\phi }^{*}}{{\phi}_{c}}\right)$$

This constitutive relationship assures that in the fully hydrated state, where ${l}_{m}=({\phi }^{*}/{\phi}_{c}){h}_{0},{K}_{PCL}={K}_{PCL-0}$. It also assures that *K*_*PCL*_ diminishes as *l*_*m*_ diminishes in inverse proportion to increasing mucin surface-to-volume. Equation (8) yields the dehydrated partition coefficient *K*_*PCL-d*_ by assigning *l*_*m*_ the value of the dehydrated mucus thickness *l*_*m-d*_. In the dehydrated state compression of airway epithelial cells increases by the amount (Supplemental Note 2)

9 $$\Delta P=\frac{q}{{L}_{p}}-{K}_{PCL-d}\Delta {\Pi}_{gp-0}+\Delta {\Pi}_{tm-0}$$

*ASL heights following hypertonic salt aerosol deposition* Following hypertonic salt aerosol deposition, the salt ion concentration differential across the apical epithelial membrane drives osmotic water flow into the ASL, with the water layer grows according to the mass balance equation

10 $$\frac{d}{dt}\left({l}_{dil}+{l}_{PCL}\right)=u-q$$

with initial conditions ${l}_{dil}\left(t=0\right)={H}_{d}(\alpha -1)$ and ${l}_{PCL}\left(t=0\right)={l}_{PCL-d}$, where *α* = (*M*_*S*_ + *M*_*ASL*_)/*M*_*ASL*_. Here, *M*_*S*_ is the total mass of salt aerosol deposited in the particular airway generation , and *M*_*ASL*_ the water mass in the ASL of the same generation. The osmotic water velocity *u* can be expressed as

11 $$u={u}_{gp}+{u}_{s}+{u}_{tm}$$

with globular protein, salt ion, and tethered mucin contributions

12 $${u}_{gp}={L}_{p}{\Pi}_{gp}$$

13 $${u}_{s}=-{L}_{p}\left({\Pi}_{s}^{C}-{\Pi}_{s}^{ASL}\right)$$

14 $${u}_{tm}={L}_{p}{\Pi}_{tm}$$

Here,

15 $${\Pi}_{gp}={K}_{PCL}(t){\Pi}_{gp-0}\left[\frac{{H}_{0}}{H(t)}\right]$$

with the osmotic pressures in the cell cytosol and the ASL given by

16 $${\Pi}_{s}^{C}=nR{T}_{k}{C}_{s}^{C}$$

17 $${\Pi}_{s}^{ASL}=nR{T}_{k}{C}_{s}^{ASL}$$

The tethered mucin osmotic pressure is given by^27^

18 $${\Pi}_{tm}\approx -\frac{180}{{\left[ln\left(1-\frac{\zeta }{{l}_{PCL-0}}\right)\right]}^{3}}$$

and the time-varying correlation length by

19 $$\zeta \approx {l}_{PCL-0}\left[1-exp\left({\left(-\frac{180}{{\Pi}_{tm}}\right)}^{1/3}\right)\right]$$

We assume *ζ* increases on a time scale *τ*_*ζ*_ with mucus lifted off the PCL, according to the relaxation process

20 $$\zeta \left(t\right)=4.4\mu m-\left[4.4\mu m-{\zeta}_{d}\right]exp\left(-\frac{t}{{\tau}_{\zeta }}\right),0<t<{T}_{PCL}$$

up to a time (*T*_*PCL*_) when the dilute layer disappears, after which

21 $$\frac{d}{dt}{l}_{PCL}=u-q,t>{T}_{PCL}$$

with the osmotic pressures due to salt as expressed in Eqs. (16) and (17) determined using the cellular ion transport model previously described^16^ and based on the original model of Sandefur, Boucher and Elston^64^. Given the rapid molecular diffusive mixing that occurs in the ASL post deposition of the hypertonic aerosol, and the relatively slow hydration time *τ*_*H*_ of the mucus hydrogel^10^, the mucus hydrogel quickly resumes its transpiration behavior post deposition. The hydrogel thickness, *l*_*m*_(*t*), is therefore assumed to retain its dehydrated thickness over the course of the hydration process. However, for aerosols with pH above the pKa of the cysteine disulfide bridges of the mucin hydrogel network, partial dissolution of the mucus layer arrests transpiration, and thus we assume the mucus layer swells on the time scale *τ*_*H*_, as has been measured in non-evaporating conditions and found to range from 2 to 10 min depending on mucin concentration^60^. This swelling continues until, with fall of pH below the cysteine pKa, the mucus hydrogel reforms, over a time *T*_*M*_. Post *T*_*M*_, the mucus hydrogel thins on a relaxation time, *τ*, ~ 42 min as recently observed^15^. Summarizing,

at low pH,

22 $${l}_{m}\left(t\right)={l}_{m-d}$$

while at high pH,

23 $${l}_{m}\left(t\right)={l}_{m}\left(t\right)=\frac{{\phi }^{*}}{{\phi}_{c}}{h}_{0}-\left[\frac{{\phi }^{*}}{{\phi}_{c}}{h}_{0}-{h}_{d}\right]exp\left(-\frac{t}{{\tau}_{H}}\right),t<{T}_{M}$$

24 $${l}_{m}\left(t\right)={h}_{d}-\left[{h}_{d}-{l}_{m}\left({T}_{M}\right)\right]exp\left(-\frac{t}{\tau }\right),t>{T}_{M}$$

Finally, we note that while for sufficiently small masses of deposited aerosol, *α* < 0.1, ASL pH remains essentially unchanged post deposition, at high aerosol masses, *α* >  > 0.1, ASL pH elevation will be significant. Given the controlled acidic nano-environment exists near the glycocalyx, partitioning of globular protein into the PCL is likely to increase, such that *K*_*PCL*-0_ > 0.7, to increasing degrees with deposited mass of the inhaled salt aerosol.

Compression of the airway epithelium Δ*P* during the rehydration and dehydration phases post hypertonic salt deposition follows as

25 $$\Delta P=\Delta {\Pi}_{gp}+\Delta {\Pi}_{tm}$$

26 $$\Delta {\Pi}_{gp}={K}_{PCL}(t){\Pi}_{gp-0}\left[\frac{{H}_{0}}{H(t)}-1\right]$$

27 $$\Delta {\Pi}_{tm}={\Pi}_{tm}-{\Pi}_{tm-0}$$

28 $${\Pi}_{tm}\approx -\frac{180}{{\left[ln\left(1-\frac{\zeta }{{l}_{PCL-0}}\right)\right]}^{3}}$$

*Airway epithelial cell compression in chronically dehydrated airways* On prolongation of the dehydrated state, or in a chronic disease state such as chronic cough, chloride flux through the apical epithelial membrane falls with down-regulation of the CFTR channel, and mucin hypersecretion leads to elevated mucus total protein (mucin and globular protein) mass fraction. In these circumstances, the following analysis permits determination of the airway epithelial cell compression with dehydration.

Assume an initial condition of the dehydrated state as characterized by Eqs. (3) to (9). With a step change fall in CFTR chloride flux into the ASL, and in the absence of a dilute layer, the thickness of the PCL falls obeying [see Eq. (10)]

29 $$\frac{d}{dt}{l}_{PCL}=u-q$$

Equations (11) through (18) apply, with the correlation length of the tethered mucin interactome in the dehydrated state determined by Eq. (5). Solving these equations with the initial conditions of Eqs. (3) through (9), for a given evaporation rate *q,* and with the CFTR activity altering the salt osmotic pressures using the cellular ion transport model previously described^16^, yields a result for the compression of airway epithelial cells using Eqs. (25) through (28) for the dilute mucin case (${\phi }^{*}<{\phi }^{c}$) considered above. At intermediate mass fractions (${\phi }^{*}>{\phi }^{c}$), the same equations apply with ${\phi }^{*}/{\phi }^{c}=1$ in Eqs. (4), (7) and (8) as also previously described^10^. At high mucin mass fractions (${\phi }^{*}>0.02$), the PCL osmotic pressure (~ 170 Pa)^15^ falls below the mucosal osmotic pressure (Π_*muc*_), such that it ceases to be determined by evaporation rate, i.e. Equation (18), and rather equals the osmotic pressure of the mucus layer. Experimental measurements of have been reported elsewhere, in the form of the individual osmotic pressures of mucin and globular proteins as a function of mass fraction of each^15^. The following empirical relationship approximates the results in the range of 0.01 < ${\phi }^{T}$  < 0.1, where ${\phi }^{T}$ is total mucosal protein (mucin and globular protein) mass fraction:

30 $${\Pi}_{muc}={\Pi}_{muc}^{*}exp\left[97.56\left({\phi }^{T}-0.01\right)\right]$$

with the asterisk referring to the mucosal osmotic pressure at ${\phi }^{T}$ = 0.01 (1.3 Pa), and recognizing that mucin to globular protein ratio remains fixed at 1:2 as mucin concentration increases. An unknown in the analysis that permits determination of the compression Δ*P* of airway epithelial cells [Eq. (25)], is the degree of sequestration of globular protein in the dehydrated mucin interactome, *K*_*PCL*_. Assuming sequestration increases significantly from the 0.3 value in the fully hydrated state, and recognizing that the osmotic pressure of the tethered mucin in the PCL becomes the predominant osmotic pressure in the PCL in this highly concentrated mucus scenario, the value of *K*_*PCL*_ in this highly concentrated mucus case can be assumed to be unity. This allows *Π*_*tm*_ to be equated to Π_*muc*_ as given by Eq. (30) [replacing Eq. (18)], and the correlation length determining the separation distance between tethered mucin follows from Eq. (19), with the thickness of the PCL by Eq. (5). The compression Δ*P* of airway epithelial cells in the concentrated case then follows from Eqs. (25) to (29).

*Numerical simulations *Fig. 2B, C, E, F, G**.** The mass transfer problem defined by Eqs. (2–24) can be solved to determine the overall height of the ASL as a function of time. The results summarized in Fig. 2 were obtained by solving the equations numerically using MATLAB (R2022a; The MathWorks, Inc., Natick, MA). The computer code is available at https://doi.org/10.5281/zenodo.21920748.

*Statistics* Statistical significance for differences in gene expression and cough bout frequency was assessed using two-sample Welch’s t-tests, assuming unequal variances between groups. Cough bout data were normalized to baseline values to account for inter-subject variability. A p-value < 0.05 was considered statistically significant. All analyses were performed using Microsoft Excel (Microsoft 365, version 2408).

## Supplementary Information

Supplementary Information.

## Data Availability

Source data for all of the figures presented in this article are freely accessible as follows. Access to the numerical code for the general of Fig. 2 will be made available on request from the communicating author. The source data for the gene expression and ASL height data shown in Fig. 4 are provided here. The source data for the human clinical data shown in Fig. 5 are provided provided here.
