# Supplementary material for: Cough hypersensitivity risk increases during heatwaves and can be reduced by inhalation of alkaline aerosols of endogenous ions
Source: Sci Rep. 2026 Sep 15;16:27655. doi: 10.1038/s41598-026-68025-4 (PMC13578754; doi:10.1038/s41598-026-68025-4)
Supplement: Supplementary file 1 [file 41598_2026_68025_MOESM1_ESM.pdf]

Supplemental Information

Cough hypersensitivity risk increases during heatwaves and can be reduced by inhalation of alkaline aerosols of endogenous ions

Button, B[1] Edwards, A[2] Chung, KF[3] Dang, H,[1] Gutay, M,[1] Griffin, N,[1] Gerenza, J.[1] Linying Wang,[4] Abubakar-Waziri, H[3] Bhatta, DB[5] Ausiello, DA[6,7] Li, D,[8] Alotaibi B,[9] Altassan, KK,[10] Edwards, DA[3,5,10]

[1]Department of Biochemistry and Biophysics, University North Carolina, Chapel Hill, United States

[2]Department of Biomedical Engineering, Boston University, Boston, United States

[3]National Heart & Lung Institute, Imperial College London, London, United Kingdom

[4]National Key Laboratory of Earth System Numerical Modeling and Application, Institute of Atmospheric Physics, Chinese Academy of Sciences, Beijing, China

[5]Sensory Cloud Inc., Boston, United States

[6]Massachusetts General Hospital, Boston, United States

[7]Harvard Medical School, Boston, United States

[8]Department of Earth and Environment, Boston University, Boston, United States

[9]College of Medicine, King Saud University, Riyadh, Saudi Arabia

[10]Department of Family and Community Medicine, College of Medicine, King Saud University, Riyadh, Saudi Arabia

[10]Center for Nanomedicine, Johns Hopkins University Medical School, Baltimore, United States

Supplemental Note 1. VPD Values, Paris, Madrid, Riyadh

Supplemental Note 2. Single-model simulations of historic & future VPD values

Supplemental Note 3: VPD and ΔP during human of extreme atmospheres

Supplemental Note 4. Dehydrated ASL height and compression formulas

Supplemental Note 5. Gene expression following exposure to warm dry air

Supplemental Note 6. ASL heights following HS pH 9 versus HDS pH9 deposition

Supplemental Note 7. REACH data all participants

Supplemental Note 8. ASL heights following HS rehydration with partial ENaC block

Supplemental References

Supplemental Note 1: VPD Values, Paris, Madrid, Riyadh

We determined the vapor pressure deficit (VPD) values for Paris and Madrid during the June 2026 heatwave, and historically for this same period from 1991 to 2026, using the *ERA5 hourly data on single levels from 1940 to present* of the Copernicus Climate Change Service (C3S) Climate Data Store (CDS). https://cds.climate.copernicus.eu/datasets. See Tables S1-S4.

1991-2026	Daytime Avg Temp (°C)	Daytime Avg RH (%)	Saturation vapor pressure (Ps) (Kpa)	Vapor pressure(Pv)	VPD (Ps-Pv)
June 18	21.11	63.52	2.51	1.59	0.91
June 19	20.54	62.8	2.42	1.52	0.90
June 20	20.34	66.79	2.39	1.60	0.79
June 21	20.62	63.83	2.43	1.55	0.88
June 22	20.13	63.06	2.36	1.49	0.87
June 23	20.32	60.39	2.39	1.44	0.95
June 24	21.34	58.24	2.54	1.48	1.06
June 25	21.58	61.19	2.58	1.58	1.00
June 26	21.77	61.7	2.61	1.61	1.00
June 27	21.57	64.12	2.58	1.65	0.92
June 28	21.66	61.21	2.59	1.59	1.01
June 29	21.27	59.96	2.53	1.52	1.01
June 30	21.11	63.14	2.51	1.58	0.92
**Average**	**21.03**	**62.30**	**2.49**	**1.55**	**0.94**
**SD**	**0.57**	**2.18**	**0.09**	**0.06**	**0.07**

Table S1. Vapor pressure deficit (VPD) values for Paris from June 18 to 30, 1991 to 2026.

1991-2026	Daytime Avg Temp (°C)	Daytime Avg RH (%)	Saturation vapor pressure (Ps) (Kpa)	Vapor pressure(Pv)	VPD (Ps-Pv)
June 18	17.16	54.03	1.96	1.06	0.90
June 19	17.11	54.52	1.95	1.06	0.89
June 20	17.22	53.87	1.97	1.06	0.91
June 21	17.2	54.32	1.96	1.07	0.90
June 22	17.12	53.91	1.95	1.05	0.90
June 23	17.22	53.62	1.97	1.05	0.91
June 24	17.28	54.03	1.97	1.07	0.91
June 25	17.23	54.12	1.97	1.06	0.90
June 26	17.13	53.95	1.95	1.05	0.90
June 27	17.16	53.2	1.96	1.04	0.92
June 28	17.14	53.53	1.96	1.05	0.91
June 29	17.66	54.44	2.02	1.10	0.92
June 30	17.91	54.08	2.05	1.11	0.94
**Average**	**17.27**	**53.97**	**1.97**	**1.06**	**0.91**
**SD**	**0.24**	**0.37**	**0.03**	**0.02**	**0.01**

Table S2. Vapor pressure deficit (VPD) values for Madrid from June 18 to 30, 1991 to 2026.

Paris					
**Date**	**Daytime Avg Temp (°C)**	**Daytime Avg RH (%)**	**Saturation vapor pressure (Ps) (Kpa)**	**Vapor pressure(Pv)**	**VPD (Ps-Pv)**
6/18/2026	30	48.2	4.25	2.05	2.20
6/19/2026	31.3	43	4.57	1.97	2.61
6/20/2026	29.9	53.2	4.22	2.25	1.98
6/21/2026	31.8	49.1	4.71	2.31	2.40
6/22/2026	33.7	44.4	5.24	2.33	2.91
6/23/2026	32.3	46.9	4.84	2.27	2.57
6/24/2026	36	33.4	5.95	1.99	3.96
6/25/2026	36.3	31.8	6.05	1.92	4.12
6/26/2026	33.4	38.4	5.15	1.98	3.17
6/27/2026	31.7	41.9	4.68	1.96	2.72
6/28/2026	26.9	59.1	3.55	2.10	1.45
6/29/2026	24.1	48.5	3.00	1.46	1.55
6/30/2026	24.4	48.1	3.06	1.47	1.59
**Average**	**30.91**	**45.08**	**4.56**	**2.00**	**2.56**
**SD**	**3.86**	**7.55**	**0.96**	**0.28**	**0.85**

Madrid					
**Date**	**Daytime Avg Temp (°C)**	**Daytime Avg RH (%)**	**Saturation vapor pressure (Ps) (Kpa)**	**Vapor pressure(Pv)**	**VPD (Ps-Pv)**
6/18/2026	30.1	23.6	4.27	1.01	3.26
6/19/2026	30.8	27.6	4.45	1.23	3.22
6/20/2026	31.4	33	4.60	1.52	3.08
6/21/2026	33.2	24.7	5.09	1.26	3.83
6/22/2026	33.8	20.6	5.27	1.08	4.18
6/23/2026	34	19.9	5.33	1.06	4.27
6/24/2026	33.4	22.2	5.15	1.14	4.01
6/25/2026	28	36	3.78	1.36	2.42
6/26/2026	28.4	33.9	3.87	1.31	2.56
6/27/2026	30.7	30.1	4.42	1.33	3.09
6/28/2026	30.1	30.9	4.27	1.32	2.95
6/29/2026	29.3	36.9	4.08	1.51	2.57
6/30/2026	32.3	31.3	4.84	1.52	3.33
**Average**	**31.19**	**28.52**	**4.57**	**1.28**	**3.29**
**SD**	**2.03**	**5.83**	**0.52**	**0.17**	**0.62**

Table S3. Vapor pressure deficit (VPD) values for Paris and Madrid from June 18 to 30, 2026.

Month	Daytime Avg Temp (°C)	Ps (kPa)	Daytime Avg RH (%)	Pv (kPa)	VPD (Ps-Pv)
**Jan**	**17.43**	**2.03**	**25.98**	**0.52**	**1.51**
**Feb**	**24.01**	**3.02**	**20.35**	**0.59**	**2.43**
**Mar**	**23.33**	**2.96**	**35.71**	**1.02**	**1.95**
**April**	**28.21**	**3.88**	**31.13**	**1.16**	**2.72**
**May**	**36.67**	**6.22**	**11.00**	**0.66**	**5.56**
**June**	**40.92**	**7.76**	**6.59**	**0.51**	**7.25**

Table S4. Vapor pressure deficit (VPD) values for Riyadh from January through June 2025.

Supplemental Note 2: Single-model simulations of historic & future VPD values

Background. To provide an estimate of vapor pressure deficit (VPD) exposure in urban areas across the United States in historical (1980 through 2019) and future (2060 through 2099) periods, we conducted a single-model analysis. This analysis is not a full climate-model simulation comparing the results of multiple models and can therefore only be viewed as the conclusions of this single model, which uses the Community Land Model version 5 (CLM5)[1], the land component of the Community Earth System Model (CESM) version 2.1.3[2]. CLM5 employs a subgrid approach to distinguish different land cover types within each grid, including natural vegetation, crop, urban, lake, and glacier. In this framework, natural vegetation and cropland are treated as rural land types. Urban climate processes are simulated using the CLM Urban (CLMU) parameterization scheme.[3-6] This subgrid approach allows us to focus on VPD over urban land within each grid cell, as extreme heat impacts on human health are disproportionately concentrated in urban areas due to both population distribution (approximately 80% of the U.S. population resides in urban settings) and the tendency for higher air temperatures and lower relative humidity (and thus higher VPD) compared to surrounding rural regions[7]. CLM5 and its urban parameterization have been extensively described, calibrated, and evaluated in previous studies[1,2], including assessments of land–atmosphere interactions and urban climate processes[3-6]. In this study, we rely on these established model parameterizations and do not perform additional calibration/validation. Instead, we use the model in its standard configuration to ensure consistency between historical and future simulations, while acknowledging that model biases and limitations remain. Specifically, we conducted land-only (uncoupled to the atmospheric model) simulations at high spatial resolution of 1/8 degree over the continental United States from 1980 to 2019. The meteorological forcing is provided by the hourly IM3/HyperFACETS dataset.[8] Based on the initial conditions provided by CESM, which have been spun up, we run CLM5 at a spatial resolution of 1/8 degree over the U.S. for additional 80-year spin-up runs, recycling the historical and future atmospheric forcings (which have been converted to 1/8 degree) twice, respectively. Finally, we conduct the 40-year historical experiment (1980-2019) and the future experiment (2060-2099) under the Representative

Concentration Pathway (RCP) 8.5 scenario, respectively, using the initial conditions provided by the spin-up runs. For analysis, heat waves (HW) are identified based on daily maximum air temperature (Tmax) over rural areas.[8] For each grid cell, the 90th and 75th percentiles of Tmax during the summer seasons (June, July, August) over 1980-2019 are computed, referred to as T1 and T2, respectively. A HW event is defined as a period of at least three consecutive days during which: (1) Tmax must exceed T1 for at least three consecutive days, (2) averaged Tmax over the entire event must exceed T1, and (3) Tmax on each day of the event must exceed T2. For future projections, the T1 and T2 values at each grid cell are calculated using the historical period (1980–2019) to ensure consistency. This definition is adopted from previous climate research[8,9]. While observational datasets may better capture local temperature variability in urban environments, we use climate model output for both historical and future periods to ensure a consistent framework. This approach allows us to analyze changes between baseline and projected conditions using a unified dataset with complete spatial and temporal coverage across all study locations. The model also includes an urban parameterization, enabling representation of urban land–atmosphere interactions in a physically consistent manner. We acknowledge, however, that model-based historical representations are subject to biases and have a limited ability to resolve fine-scale urban heterogeneity.

<u>Results.</u> We assessed the risk of human airway exposure to inflammatory dry air during extreme heat events over urban centers in the United States in the historical (1980 through 2019) and the future (2060 through 2099) periods for the continental United States by performing a land-only climate model simulation using the Community Land Model Urban (CLMU) parameterization scheme[48-51] (Methods). We used 1.5 to 3.0 kPa VPD to describe moderately inflammatory air, given the finding that mucosal collapse occurs within one hour on exposure to air of this aridity level (35 ºC, 60% RH),[10] and 3.0 to 4.5 kPa to describe highly inflammatory air, given the finding that on exposure to air of this aridity level (35 ºC, 30% RH), acute secretion of inflammatory cytokines occurs.[10] Our findings are shown in Figure S1.

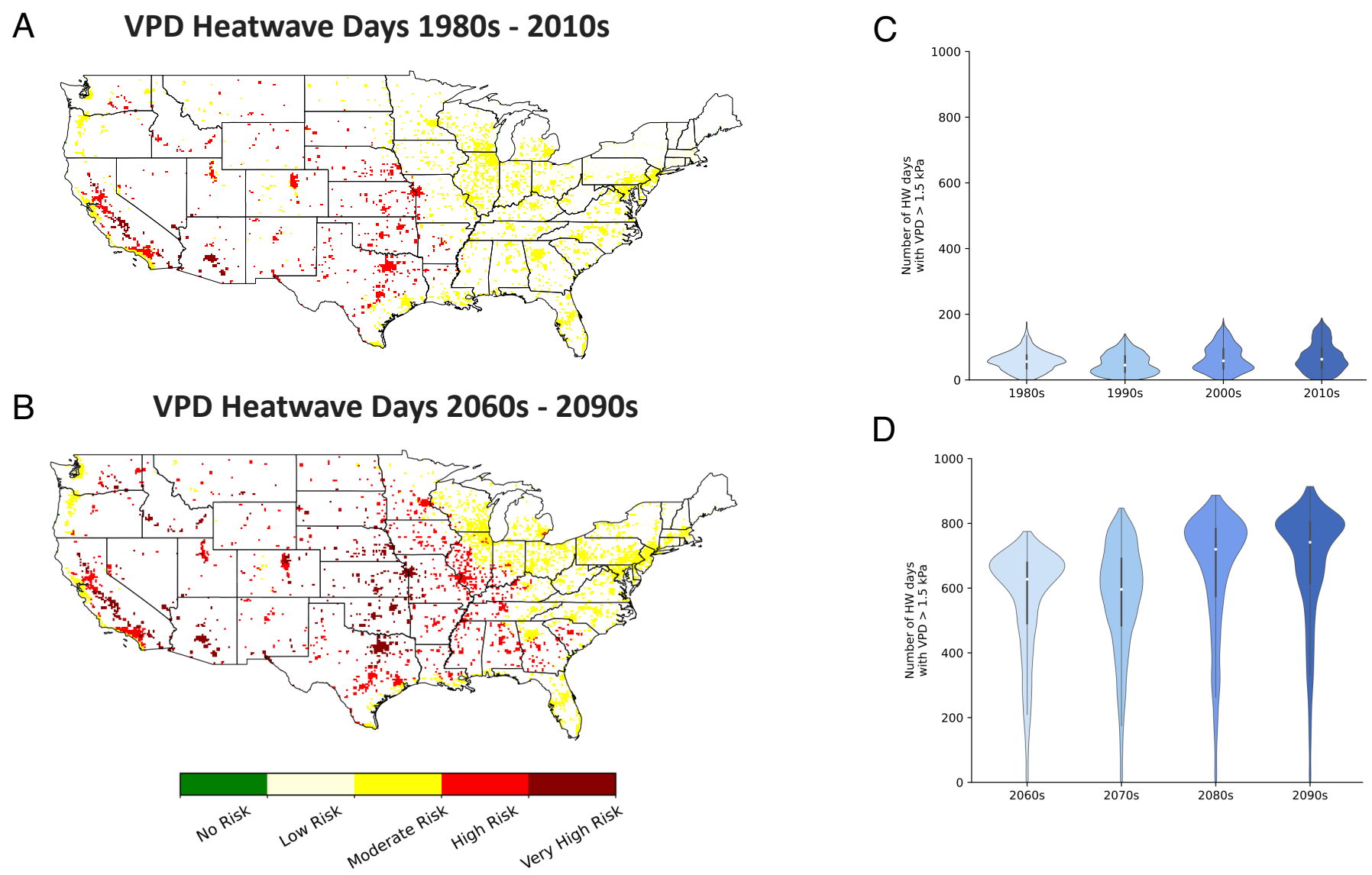

Figure S1. Extreme heat event and VPD exposure in the continental United States from 1980 to 2099. Projections of environmental (outdoor air) VPD over urban land for the continental US over the historic period 1980-2019 and future periods 2060 - 2099 based on a climate model simulation carried out using CESM version 2.1.3 as further described in the Methods. A city must be larger in terms of surface area fraction than 0.1% of the grid cell to be included. The grid cell size is about 12.5 km (1/8 degree). In total, there are 5410 cities considered here. Average VPD in summer months (June, July, August) on heat-wave (HW) days in the historic (A) and future (B) periods. Dark green denotes no risk (VPD < 750 Pa), light yellow denotes low risk (750 Pa < VPD < 1.5 kPa), dark yellow denotes moderate risk (1.5 kPa < VPD < 3.0 kPa), red denotes high risk (3.0 kPa < VPD < 4.5 kPa) and dark red denotes very high risk (VPD > 4.5 kPa). The VPD values reported here are for the urban part of the grid cell (i.e., for the urban land). (C & D) The total number of HW days with VPD > 1.5 kPa per decade summed over the simulated 5410 cities over the continental US per decade across the simulated 5410 cities over the continental US in the historic (C) and future (D) periods. The shape of each violin represents the distribution of HW days, with the internal box indicating the interquartile range and the central white dot indicating the median value. Source code available on request.

Heat-wave (HW) days during the summer season (June, July, August) shifted from mostly moderate risk in the 1980 to 2020 period (Fig SI-1A) to high risk in the 2060 to 2100 period (Fig S1B). With the exception of the 1990s, when global cooling occurred on account of the Mt Pituobo volcanic eruption in the Philippines in 1991,[52] HW days grew gradually more frequent across the historic period (Fig S1C), while are predicted to increase dramatically in frequency as the 21st century advances (Fig S1D). During the 2010s heatwaves occurred on approximately 7.6% of all summer days (375,816 of 4,977,200 days) (Fig S1C); by comparison, the percentage

of summer days that will be HW days in the 2060s is predicted to be 61.4%, in the 2070s 61.7%, in the 2080s 70.8% and in the 2090s 73.8% (Fig S1D). The total number of HW days with VPD > 3 kPa also increases dramatically, from 155,501 in the 2010s to 1,460,409 in the 2060s. By the 2090s, HW days with VPD > 3.0 kPa (1,955,980) are predicted to increase 12.6-fold relative to the 2010s. Notwithstanding global warming, non-heatwave (non-HW) days (not shown) remain mostly at no-risk or low-risk in the historic and future periods.

	**Airway VPD on mouth breathing 20-25 °C air, 35% RH (1.5 kPa VPD)**							**Mean airway values**			
Generation	0	1	2	3	4	5	6	**0-6 gen**	**0-5 gen**	**0-4 gen**	**0-3 gen**
RH (%)	40	65	75	83	89	94	98				
VPD (kPa) @35°C	3.38	1.97	1.41	0.96	0.62	0.34	0.11	1.25	1.44	1.67	1.93
q (m/s)	3.51E-07	1.48E-07	1.06E-07	7.20E-08	4.66E-08	2.54E-08	8.47E-09	1.08E-07	1.25E-07	1.45E-07	1.69E-07
	**Airway VPD on mouth breathing 40 °C air with 15% RH (6.0 kPa VPD)**							**Mean airway values**			
Generations	0	1	2	3	4	5	6	**0-6 gen**	**0-5 gen**	**0-4 gen**	**0-3 gen**
RH (%)	20	50	65	75	83	89	95				
VPD (kPa) @40°C	5.91	3.69	2.58	1.85	1.26	0.81	0.37	2.35	2.68	3.06	3.51
q (m/s)	5.00E-07	2.26E-07	1.58E-07	1.13E-07	7.69E-08	4.98E-08	2.26E-08	1.64E-07	1.87E-07	2.15E-07	2.49E-07

Table S5. Evaporation rates and VPD for human airways in a mild and extreme heat wave circumstance. Humidity values for airway generations 0-6 are obtained following (1). Mouth/pharyngeal heating of 20-25°C air to 35°C (Case A) and no heating of 40°C air (Case B) is assumed following (2). Pharyngeal humidification is assumed to add 5% RH at the glottal air temperature on inhalation. VPD is determined at the given relative humidity and temperature as the difference between water saturation pressure and the actual vapor pressure using the Clausius-Clapeyron Equation (3). Absence of the laryngeal jet outside of the larynx and trachea reduces the convective (ventilation rates V_R) contribution to Eq (1) in subsequent airways by a factor of 2.

HW days over all cities were unevenly distributed, as illustrated by the shapes of the violin plots in Figs 1C and 1D. Cities with greater than 125 HW Days in the 1980s and 90s (165) multiplied 7.7 fold by the 2000s and 2010s (1271), while cities with greater than 125 HW days in the 2010s (780) are predicted to increase 6.8 fold in the 2090s (5,331). Cities with greater than 750 HW days increase from 0 in the 2010s to 556 in the 2060s, 1,438 in the 2070s, 3,422 in the 2080s and 4,212 in the 2090s. In terms of the severity of heat stress experienced by the US population on

these HW days, average VPD in the ten most populous US cities (New York City, Los Angeles, Chicago, Houston, Phoenix, Philadelphia, San Antonio, San Diego, Dallas, Jacksonville) remained consistently ~ 2.7 kPa across the four decades 1980-2020, and ~ 6.0 kPa in the ten hottest cities. Using this same model, and assuming a mean global temperature rise of 2.7ºC by 2100, we find that, across the future period 2060-2100, mean VPD for the 10 largest cities increases to ~ 3.4 kPa, and mean VPD in the ten hottest cities increases to ~ 7.0 kPa.

Supplemental Note 3. VPD and ΔP during human of extreme atmospheres

Table S5 summarizes evaporation rates based on airway VPD exposures for larynx/trachea (generation 0), primary/secondary/tertiary bronchi (generations 1, 2, 3) and first brionchiole (generations 5, 6) airways using Eq (1) of the main manuscript. The base and extreme cases of the main manuscript are considered: tidal human mouth breathing of (A) indoor cooled air during a heat wave, characterized by indoor air with VPD = 1.5 kPa, and (B) outdoor hot air with VPD = 6.0 kPa. With the characteristic mucin diffusivity D = 2x10^{-12} m^2/s and mucus thickness h_0 = 2x10^{-5} m, the average characteristic Pe (=qh_0/D) ranges from 1 to 1.6 in generations 0 to 6 to 1.9 to 3.5 in generations 0 to 3. Extremely high VPD (3.4 to 5.9) in the larynx/trachea in the A & B cases results in ultra-high Pe (3.5 to 5) highlighting the risks of laryngeal/tracheal inflammation in cooled indoor and hot outdoor conditions commonly encountered during heat waves.

For the indoor air "mild" heatwave condition (A), the average partitioning of globular protein into the PCL over the full airway generation range 0 to 6 (Pe ~ 1) diminishes [see Eq (8) and Eq (9) of Methods] from 0.7 to 1.44 (VPD = 1.5 kPa), such that, ΔP = 0.98 kPa. This is the base dehydrated case on which the simulations in Fig 2 are based. In the extreme case B, over generations 0 to 4 (Pe ~ 3, VPD ~ 3.0 kPa), greater mucus thinning and sequestering of globular protein results in K_{PCL-d}= 0.28, with ΔP = 2.39 kPa.

It is further instructive to consider the case of slow nasal breathing, for which, with Pe ~ 0.2, L_p ~ 0.8x10^{-10} m^2-s/kg, K_{PCL-d} ~ 0.7, and Π_{gp-0} = 350 Pa, the globular protein osmotic water velocity is given by Eq (12) and Eq (15) of the Methods as u_{gp} ~ 0.026 μm/s. Similarly, with Π_{tm} ~ 180

Pa, Eq (8) of the Methods gives u_{tm} ~ 0.014 μm/s. Noting that $u_s = 0$ in the steady state, the net osmotic water velocity u ~ 0.04 μm/s exceeds the rate of evaporation q ~ 0.02 μm/s, such that the ASL hydrates, and dilutes globular protein concentration sufficiently to reach water homeostasis. Increasing evaporation rate by 100% from q ~ 0.02 μm/s to 0.04 μm/s, as occurs on normal tidal nasal breathing (V_R ~ 15 L/min), thins the mucus hydrogel, while increasing the globular protein partitioning into the hydrogel, such that according to Eq (11) the water velocity through the hydrogel remains roughly constant at ~ 0.024 μm/s. Thus the overall osmotic water velocity u ~ 0.04 μm/s equals the evaporation rate, indicating that the base $\Pi_{gp\text{-}0} = 350$ Pa is appropriate to support water homeostasis during nasal tidal breathing at a normal tidal ventilation rate.

Supplemental Note 4. Dehydrated ASL pressure elevation

In the steady dehydrated state, which a time-average view of the ASL over the course of the continuous breathing of air of the same atmospheric condition for approximately one minute or more — salt ions equilibrate. The average net flow of water from the airway epithelium into the ASL to support the net evaporation of water into the air lumen is entirely supported by the osmotic pressures of globular protein and tethered mucin. This gives the water mass balance

$$q = u_{gp} + u_{tm}$$

Using Eq (15) and Eqs (3)-(6) of Methods, it follows that the globular protein contribution to the osmotic water velocity is

$$u_{gp-d} = L_p K_{PCL-d} \Pi_{gp-0} \left(\frac{H_0}{H_d}\right) \approx L_p K_{PCL-d} \Pi_{gp-0} \left(\frac{h_0}{h_d}\right) \approx \frac{\frac{Pe_0}{3} L_p K_{PCL-d} \Pi_{gp-0}}{\left[\left(1+\frac{4}{3}Pe_0\right)^{1/4} - 1\right]}$$

The tethered-mucin contribution to the water velocity further follows from $u_{tm\text{-}d} = L_p\, \Pi_{tm\text{-}d}$, and with

$$\Pi_{tm-d} \approx \frac{q}{L_p} - \frac{\frac{Pe_0}{3} K_{PCL-d} \Pi_{gp-0}}{\left[\left(1+\frac{4}{3}Pe_0\right)^{1/4} - 1\right]}$$

The total pressure elevation [Eq (1) of the main article and Eq (25) of Methods] is then simply

$$\Delta P_d = \frac{q}{L_p} - K_{PCL-d}\Pi_{gp-0} - \Pi_{tm-0}$$

Supplemental Note 5. Gene expression following exposure to warm dry air

Figure S2 summarizes gene expression results obtained following exposure of ALI cultures of HBE cells (Methods) to an extreme atmosphere of 4.4 kPa for 8 hours in a humidity-controlled confocal microscope chamber with air circulation: ANO1, AQP1, AQP5, CFTR, F2R1, ORAl1, P2RX3, P2RX4, P2RX7, P2RY2, PIEZO1, PIEZO2, SCNN1A, SCNN1B, SCNN1D, SCNN1G, TRPA1, TRPV1, TRPV2, TRPV3, TRPV4, TRPV5, TRPV6. All genes up-regulated at either 3h or 8h or both with the exception of AN01, CFTR, F2RL1, ORAl1, P2RX4, P2RY2, SCNN1G, TRPV1. Genes associated with chloride channel activity ANO1, CFTR (Fig S2A) down-regulate, cough provocation — notably the P2X3 cough receptor gene (Fig 21B) — up-regulate, sodium-channel activity — notably the α and β units of ENaC (SCNN1A, SCNN1B) (Fig S2C) up-regulate, TRPV cough activation — notably TRPV1 (Fig S2D) — down-regulate, and ATP secretion — notably TRPV4 (Fig S2D) — up-regulate.

Supplemental Note 6. ASL heights following HS pH 9 versus HDS pH9 deposition

Figure S3 presents the results of our numerical simulations of topical deposition of alkaline (pH 9) HS (NaCl) and HDS ($MgCl_2$) aerosols on dehydrated airway mucosa following the circumstances of Fig 2B of the main article for non-alkaline aerosol deposition. Unlike the non-alkaline case of Fig 2B, water uptake in the mucus layer is significant given the disassociation of the mucus hydrogel caused by high pH. The benefit of the slow clearance of the divalent magnesium cation is to significantly prolong hydration (dotted red line) relative to the monovalent sodium cation (red solid line).

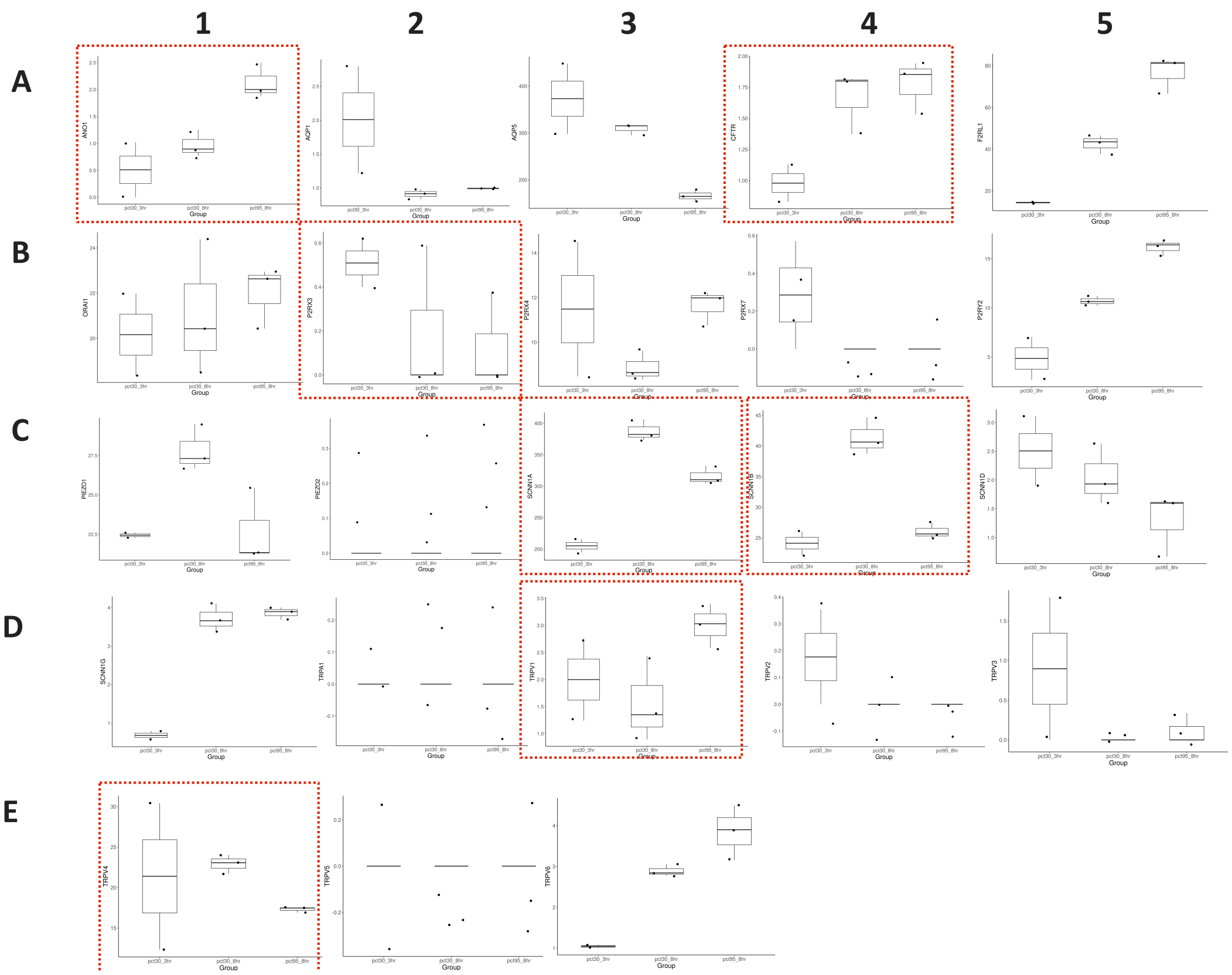

Figure S2. Gene expression following 3 to 8 hours of exposure to 37°C, 30% RH (VPD = 4.4 kPa) air. Red boxes highlight notable airway/cough hypersensitivity gene expression findings.

Supplemental Note 7. Additional information from the REACH trial

Active and placebo. The REACH trial evaluated the safety and efficacy of SC0023, an alkaline HDS with pH 9, comprised of 4.3 % magnesium chloride, 0.015% sodium bicarbonate and 0.086% sodium carbonate. SC0023 produces a mass-median-aerodynamic diameter (MMAD) of approximately 10 μm on delivery from the soft-mist pump spray (Ursatec, Tholay, Germany), in comparison to an MMAD of approximately 23 μm produced by the normal saline placebo control delivered from the same device.

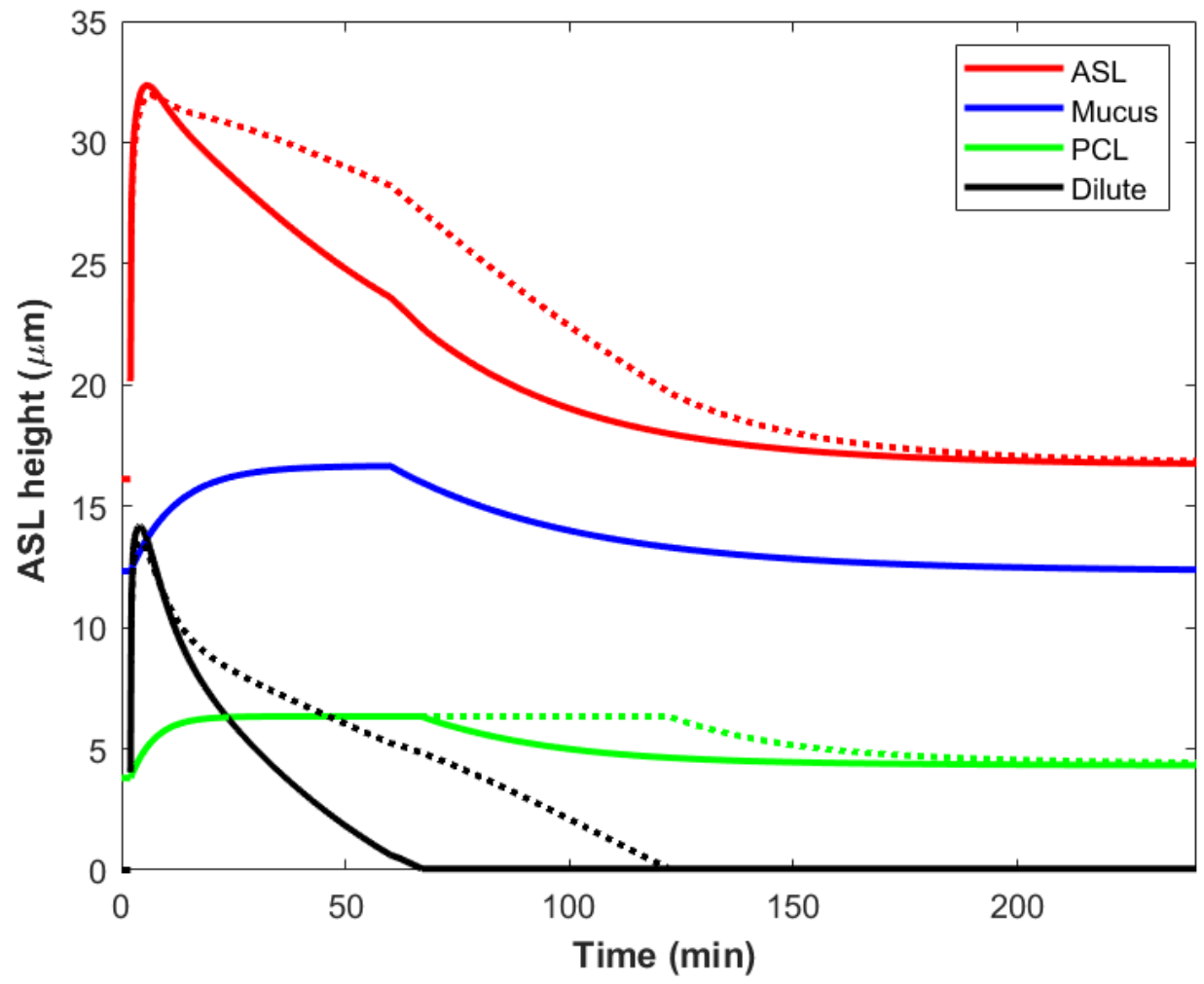

Figure S3. Rehydration of dehydrated airway mucus following deposition of HS & HDS pH 9 aerosols. Change in the heights of ASL, mucus hydrogel, dilute mucin layer, and PCL vs time post deposition of HS (NaCl, 4.3% w/w, pH 9) (solid lines) and HDS ($MgCl_2$, 4.3% w/w, pH 9) (dashed lines) with total deposited mass 25% of ASL mass. Airway generations 0 to 6 in normal tidal breathing (15 L/min), or generations 0 to 9 with fast breathing (30 L/min), are assumed dehydrated by the mouth breathing of 25°C, 30% RH air, resulting in exposure to an average VPD ~ 1.5 kPa (ranging from ~ 3.4 kPa in the larynx to ~ 0.5 kPa in the first bronchioles).

Variable		Active	Placebo
**Age (years)**	Mean ± SD	42.4 ± 17.1	51.2 ± 14.5
	Median (Q1 - Q3)	45 (26 - 57.5)	58 (37 - 62)
	Min - Max	23 - 66	29 - 66
**Sex, n (%)**	Female	1 (20.0%)	3 (60.0%)
	Male	4 (80.0%)	2 (40.0%)
**BMI* (Kg/m^2)**	Mean ± SD	31.5 ± 9.6	26.8 ± 7.9
	Median (Q1 - Q3)	27.8 (26.4 - 38.5)	28.6 (19 - 33.8)
	Min - Max	24.9 - 48.5	16.8 - 36.8

*BMI: body mass index.

Table S6: Participants' demographics, anthropometrics, and vital signs (N = 10)

Intention-to-treat (ITT) and per-protocol (PP) populations. Table S6 summarizes the demographics and vital signs of the intention-to-treat (ITT) participants. Table S7 summarizes the ITT (n=10) and per-protocol (PP) (n=7) primary outcome variables.

Post-hoc population. Upon unblinding, measurement of inhaler weights indicated that one of the participants received less than a single administration over the two-week treatment period. This participant was excluded in the post-hoc analysis. Table S8 summarizes primary outcome variables from the post-hoc analysis. Figure S4 presents the daily cough and cough bout data per participant over baseline (1 week), treatment (2 week), and post treatment (1 to 3 week) periods.

Variable	Population	Treatment group	n	Mean ± SD	p-value	
					T-test	Nonparametric
%E	ITT	Active	5	31.8 ± 38.4	0.702	0.310
		Placebo	5	24.7 ± 11.0		
%E	PP	Active	4	32.8 ± 44.3	0.637	0.400
		Placebo	3	19.5 ± 6.8		

%E: placebo-adjusted efficacy of cough frequency reduction; ITT: intention to treat; PP: per protocol; SD: standard deviation

Table S7: Primary outcome analysis for ITT and PP populations

Variable	Population	Treatment group	n	Mean ±SD	p-value	
					T-test	Nonparametric
%E	Case 4d	Active	3	53.5 ±19.2	0.045*	0.100
		Placebo	3	19.5 ± 6.8		0.050*†

*Statistically significant
†One-sided p-value
d PP excluding patient with apparently less than single dose in first treatment period
%E: placebo-adjusted efficacy of cough rate reduction; PP: per protocol; SD: standard deviation

Table 8: Primary outcome analysis for the post-hoc PP population

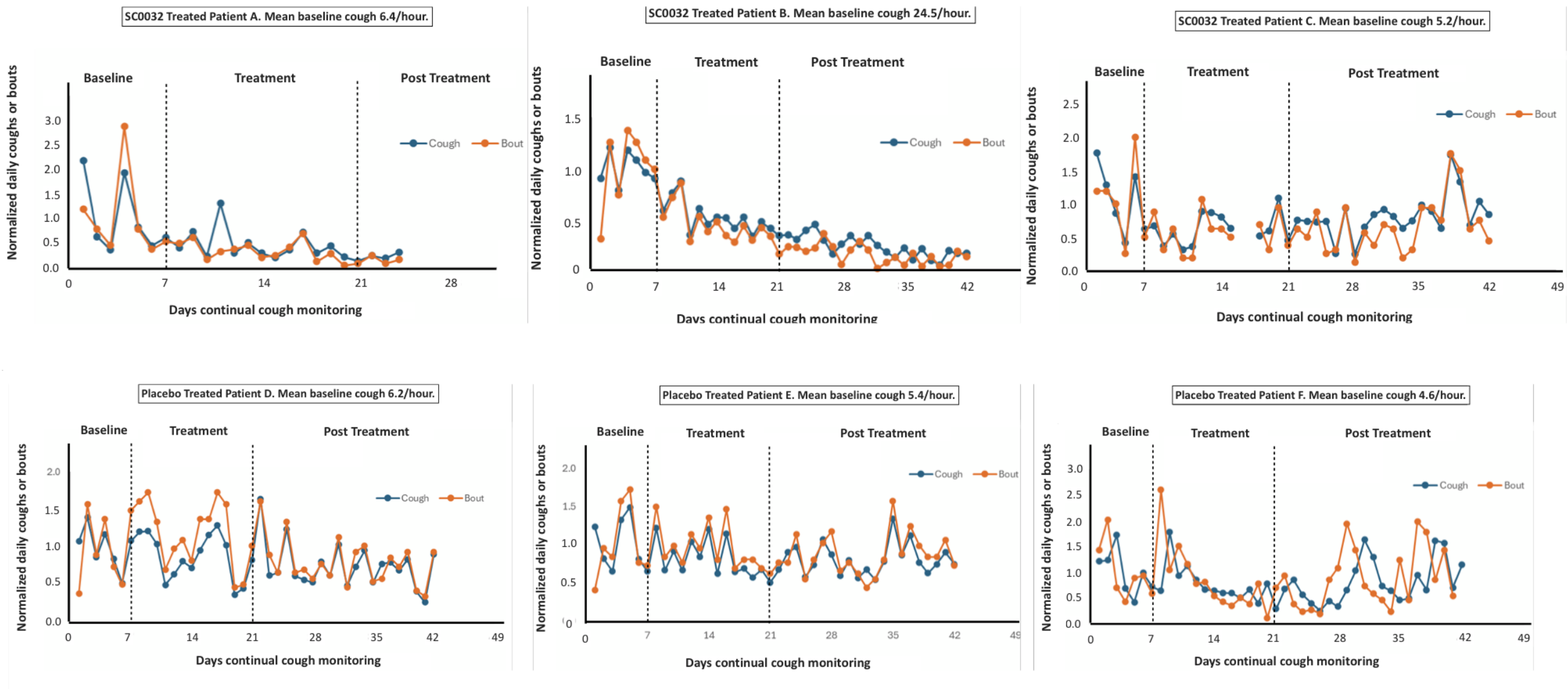

Figure S4. Daily cough and cough bout counts as measured by the Hyfe digital cough monitor. Monitoring occurred over one week baseline cough assessment, two weeks active or placebo treatment, and one to three weeks post-treatment for active-treated (n=3) and placebo-treated (n=3) RCC subjects in the REACH trial.

Supplemental Note 8. ASL heights following HS rehydration with partial ENaC block

Figure S5 presents the results of our numerical simulations of the effect of a 15% block of ENaC on ASL height evolution following the deposition of 4.3% NaCl pH 7 and pH 9. Minor prolongation of hydration is achieved by the 15% ENaC block relative to the hydration prolongation observed experimentally in Fig 4F of the main article with the pH 9 hypertonic aerosol relative to pH 7.

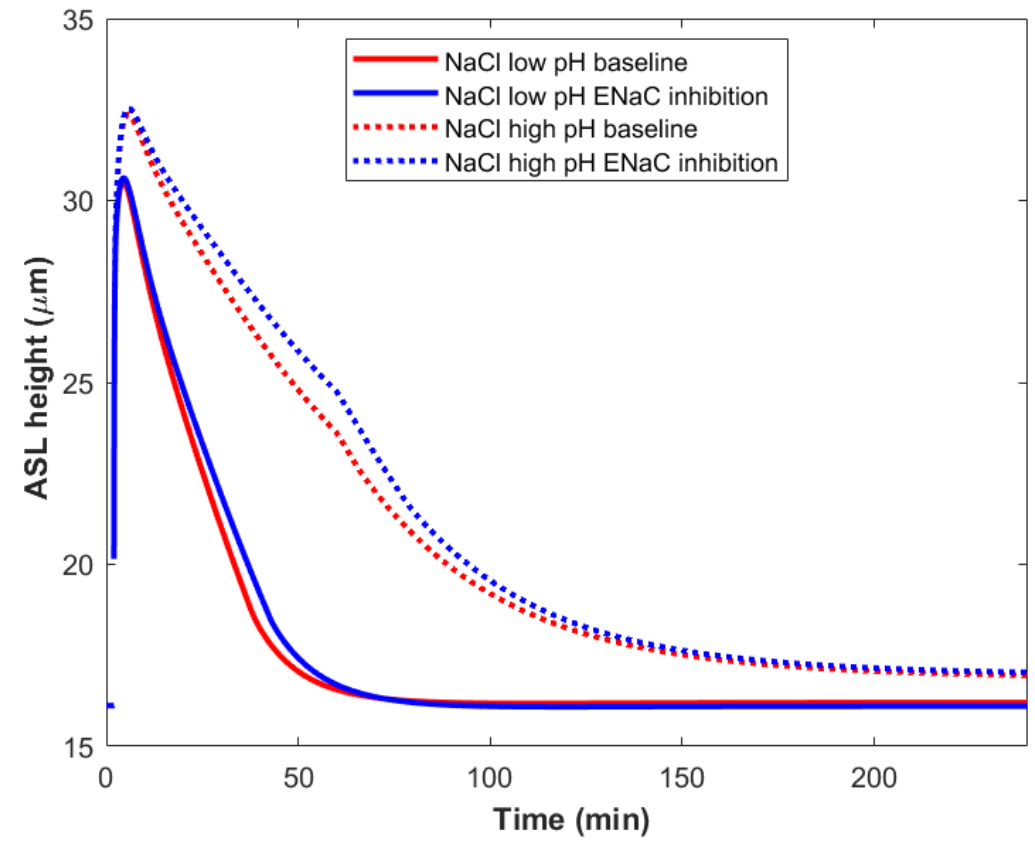

Figure S5. Rehydration of dehydrated airway mucus following deposition of HS & HDS pH 9 aerosols. Numerical simulations of ASL height post deposition of HS (4.3% NaCl w/w) pH 7 (solid lines) and pH 9 (dashed lines) aerosols with normal ENaC activity (red lines) and 15% ENaC block (dashed lines).

References

1. Lawrence, D. M. et al. The community land model version 5: description of new features, benchmarking, and impact of forcing uncertainty. *J. Adv. Model. Earth Sys.* **11**, 4245–4287 (2019).
2. Danabasoglu, G. et al. The community earth system model version 2 (CESM2). *J. Adv. Model. Earth Sys.* **12**, e2019MS001916 (2020).
3. Oleson, K. W., Bonan, G. B., Feddema, J., Vertenstein, M. & Grimmond, C. S. B. An urban parameterization for a global climate model. Part I: formulation and evaluation for two cities. *J. Appl. Meteorol. Clim.* **47**, 1038–1060 (2008).
4. Oleson, K. W., Bonan, G. B., Feddema, J. & Vertenstein, M. An urban parameterization for a global climate model. Part II: Sensitivity to input parameters and the simulated urban heat island in offline simulations. *J. Appl. Meteorol. Climatol.* **47**, 1061–1076 (2008).
5. Oleson, K. W., Bonan, G. B., Feddema, J., Vertenstein, M. & Kluzek, E. *Technical description of an urban parameterization for the Community Land Model (CLMU).* NCAR Technical Note NCAR/TN-481+STR, National Center for Atmospheric Research, Boulder, CO (2010).

6. Oleson, K. W. & Feddema, J. Parameterization and surface data improvements and new capabilities for the community land model urban (CLMU). *J. Adv. Model Earth Sys.* **12**, e2018MS001586 (2020).
7. Jones, A. D. et al. Continental united states climate projections based on thermodynamic modification of historical weather. *Sci. Data* **10**, 664 (2023).
8. Lau, N.-C. & Nath, M. J. Model simulation and projection of European heat waves in present-day and future climates. *J. Clim.* **27**, 3713–3730 (2014).
9. Wang, L., Li, D. & Yuan, X. The role of vapor pressure deficit in the CLM simulated interaction between urban heat islands and heat waves over CONUS. *Geophys. Res. Lett.* **52**, e2024GL113257 (2025).
10. Hill DB, Button B, Rubinstein M, Boucher RC. Physiology and pathophysiology of human airway mucus. Physiol Rev. **102,** 1757-1836 (2022).
11. Edwards DA, Edwards, A, Li, D Wang, L, Chung, KF, Bhatta, D, Bilstein, A Hanes,, Endirisinghe, I, Burton Freeman, B, Button, B. Global warming risks dehydrating and inflaming human airways. Nature Commun Earth Environ 6, 193 (2025).
12. Sandefur CI, Boucher RC, Elston TC. Mathematical model reveals role of nucleotide signaling in airway surface liquid homeostasis and its dysregulation in cystic fibrosis. Proc Natl Acad Sci U S A. 2017 Aug 29;114(35):E7272-E7281.

13. Dobin A, Davis CA, Schlesinger F, Drenkow J, Zaleski C, Jha S, Batut P, Chaisson M, Gingeras TR. STAR: ultrafast universal RNA-seq aligner. Bioinformatics. 2013 Jan 1;29(1):15-21
14. Shumate A, Wong B, Pertea G, Pertea M. Improved transcriptome assembly using a hybrid of long and short reads with StringTie. PLoS Comput Biol. 2022 Jun 1;18(6):e1009730.
15. Law CW, Chen Y, Shi W, Smyth GK. voom: Precision weights unlock linear model analysis tools for RNA-seq read counts. Genome Biol. 2014 Feb 3;15(2):R29.

16. Ritchie ME, Phipson B, Wu D, Hu Y, Law CW, Shi W, Smyth GK. limma powers differential expression analyses for RNA-sequencing and microarray studies. Nucleic Acids Res. 2015 Apr 20;43(7):e47.

17. Oleson, K. W., Bonan, G. B., Feddema, J., Vertenstein, M. & Grimmond, C. S. B. An urban parameterization for a global climate model. Part I: formulation and evaluation for two cities. *J. Appl. Meteorol. Clim.* **47**, 1038–1060 (2008).

18. Oleson, K. W., Bonan, G. B., Feddema, J. & Vertenstein, M. An urban parameterization for a global climate model. Part II: Sensitivity to input parameters and the simulated urban heat island in offline simulations. *J. Appl. Meteorol. Climatol.* **47**, 1061–1076 (2008).

19. Oleson, K. W., Bonan, G. B., Feddema, J., Vertenstein, M. & Kluzek, E. *Technical description of an urban parameterization for the Community Land Model (CLMU).* NCAR Technical Note NCAR/TN-481+STR, National Center for Atmospheric Research, Boulder, CO (2010).

20. Oleson, K. W. & Feddema, J. Parameterization and surface data improvements and new capabilities for the community land model urban (CLMU). *J. Adv. Model Earth Sys.* **12**, e2018MS001586 (2020).

21. McCormick, M., Thomason, L. & Trepte, C. Atmospheric effects of the Mt Pinatubo eruption. *Nature* **373**, 399–404 (1995).
